# Observability- and Identifiability-Guided Sensor-Set Design for Digital-Twin-Assisted Consolidated Bioprocessing

**DOI:** 10.3390/s26123948

**Published:** 2026-06-21

**Authors:** Mark Korang Yeboah, Nana Yaw Asiedu, Ahmad Addo

**Affiliations:** 1 Chair of Dynamics and Control, University of Duisburg–Essen, Lotharstraße, 47057 Duisburg, Germany; 2 Faculty of Mechanical and Chemical Engineering, Kwame Nkrumah University of Science and Technology, PMB, University Post Office, Kumasi 00233, Ghana

**Keywords:** consolidated bioprocessing, digital twin, sensor-set design, observability analysis, parameter identifiability, fisher information matrix, soft sensing, unscented Kalman filter, hybrid gray-box model, measurement uncertainty

## Abstract

Consolidated bioprocessing (CBP) is difficult to monitor because enzyme production, lignocellulose degradation, sugar release, and fermentation occur simultaneously under sparse measurement, feedstock variability, and plant–model mismatch conditions. This study proposes a computational sensor-set design framework for digital-twin-assisted CBP monitoring. A five-state virtual plant, consisting of active biomass, cellulolytic enzyme activity, residual insoluble substrate, soluble sugar, and ethanol, was used to evaluate all 16 ethanol-mandatory measurement packages formed from ethanol, sugar, biomass, enzyme, and residual-substrate proxy channels. Candidate sensor sets were assessed using finite-difference output sensitivities, Fisher-information-based state-observability and parameter-identifiability analyses, eigenvalue and parameter-correlation diagnostics, and paired Monte Carlo unscented Kalman filter soft-sensing reconstruction. Within the tested five-state virtual-plant benchmark and with the specified excitation schedule, noise assumptions, burden indices, and scoring objective, ethanol-only sensing provided the weakest support for state-aware CBP digital-twin reconstruction. At a 6h sampling interval, the state-observability log-pseudodeterminant increased from 4.18 with ethanol-only sensing to 8.56 after adding soluble sugar and to 16.42 with full-proxy monitoring. The ethanol–sugar–biomass–substrate package also gave strong reduced state-observability performance, with log-pseudodeterminants of 15.12, 13.76, and 12.51 at 6, 12, and 24h, respectively. Biomass and enzyme proxies contributed strongly to parameter learning, and the ethanol–sugar–biomass–enzyme package gave the strongest active parameter-identifiability performance, with log-pseudodeterminants of 10.82, 9.06, and 6.67 at 6, 12, and 24h, respectively. In the paired soft-sensing analysis, full-proxy monitoring reduced the mean latent-state RMSE from 1.1899 to 0.3756, followed by ethanol–biomass–enzyme–substrate with 0.3843 and ethanol–sugar–biomass–substrate with 0.4121. The primary aggregate ranking identified ethanol–sugar–biomass–substrate as the best overall package, with a sensor-value score of 0.8432 and a burden index of 7.0, followed by full-proxy monitoring with a score of 0.8173 and a burden index of 10.0. Robustness tests showed that ethanol–sugar–biomass–substrate remained top-ranked under uniform noise scaling, full UKF missingness, delay and bias stress test conditions, most scoring-weight scenarios, and all tested sensor-specific burden workflows. Full-proxy monitoring remained a close competitor under independent sensor-specific noise variation conditions and became top-ranked for some alternative operating trajectories. The proposed framework provides a simulation-based method for prioritizing informative measurement packages before implementing CBP digital twins in laboratory and pilot-plant settings.

## 1. Introduction

Consolidated bioprocessing (CBP) is a process-intensified route for producing ethanol and other biochemical products through the simultaneous biological integration of enzyme production, biomass deconstruction, and fermentation [1,2]. Its appeal lies in the potential to reduce dependence on externally supplied cellulases and to simplify the biomass-to-products chain compared with separated enzyme production, hydrolysis, and fermentation schemes. However, CBP remains technically challenging because its performance depends on host engineering, cellulosome biosynthesis, microbial consortia design, enzyme delivery, substrate accessibility, and feedstock deconstruction. Recent studies have therefore focused on CBP strains and genetic manipulation, synthetic cellulosomes and extracellular polymeric substances, microbial communities and co-cultures, enzyme delivery strategies, substrate accessibility, and process intensification [3,4,5,6,7]. From a monitoring perspective, CBP is not only a biochemical conversion process whose product yield should be maximized; it is also a nonlinear, partially observed dynamic system in which growth, enzyme synthesis, insoluble-substrate deconstruction, sugar release, sugar consumption, fermentation, and inhibition-related effects evolve on different time scales.

A central difficulty in developing digital-twin-assisted CBP is the limited availability of informative online measurements. Ethanol concentration is often one of the most accessible measurements, but it is a delayed product signal and cannot directly reveal whether poor batch performance originates from weak biomass growth, insufficient enzyme production, poor substrate accessibility, slow hydrolysis, sugar limitation, or product inhibition. In contrast, states that are more useful for process decision-making, such as living biomass concentration, active enzyme concentration, residual insoluble substrate, and soluble sugar concentration, are difficult to measure directly, continuously, or non-invasively. Similar measurement limitations have motivated the use of hybrid models, soft sensors, and online state-estimation methods in bioprocess monitoring and control [8,9,10,11]. Recent studies have also shown the value of soft-sensor recalibration, metabolic-heat-based soft sensing, spectroscopic monitoring, real-time biomass estimation, and Kalman-filter-based state–parameter estimation for improving the information content of bioprocess measurements [12,13,14,15,16,17,18]. In parallel, digital bioprocessing and digital chemical engineering studies emphasize accurate process measurements, model integration, predictive modeling, enabling digital technologies, and the progressive introduction of process analytical technology tools during process development [19,20,21,22,23,24,25]. For nonlinear systems, unscented Kalman filtering provides a convenient way to propagate uncertainty through nonlinear dynamics without local linearization [26,27]; therefore, the quality of soft sensing depends strongly on the informativeness of the available measurements.

The problem of choosing measurements for CBP cannot be reduced to the selection of a state-estimation algorithm. It is also necessary to determine whether the available measurements contain enough information for state reconstruction and parameter learning. State observability describes the extent to which hidden process states can be reconstructed from measured outputs, whereas parameter identifiability describes the extent to which model parameters can be estimated from available data. These concepts are especially important in partially observed biochemical systems, where unmeasured states and uncertain parameters can compensate for one another and produce similar measured trajectories [28,29]. Fisher-information- and sensitivity-based metrics provide practical tools for quantifying measurement informativeness, observability, and identifiability. This issue is particularly important for CBP because candidate measurements differ substantially in measurement burden, cost, delay, and online implementation difficulty. For example, ethanol, soluble sugar, biomass proxies, enzyme-activity proxies, and residual-substrate proxies are not equally easy to obtain; therefore, their value for digital-twin deployment should be evaluated before laboratory or pilot-scale implementation. Recent literature-derived CBP modeling has also shown that product prediction is strongly affected by heterogeneous feedstock–pretreatment–microbial descriptors, sparse product reporting, and missing-label structure [30].

Although CBP has been widely studied from biological, biochemical, and process-intensification perspectives [3,6,7,31,32], systematic evaluation of measurement sets for CBP digital twins remains limited. Existing soft-sensing and bioprocess-control literature demonstrates that software sensors and nonlinear state-estimation methods can improve process monitoring [8,9,10,11]. More recent work has further highlighted the importance of soft-sensor generalizability, sensor recalibration, biomass monitoring, spectroscopic data streams, and joint state–parameter estimation for reliable model-assisted monitoring [12,13,14,16,17,18]. However, there is still no systematic comparison of CBP measurement packages with respect to state observability, parameter identifiability, soft-sensor reconstruction performance, measurement burden, and robustness to measurement uncertainty and alternative scoring priorities. In particular, it remains unclear whether ethanol-only sensing is sufficient for constructing a state-aware CBP digital twin, whether ethanol and sugar measurements provide an adequate minimal package, or whether additional biomass, enzyme, and substrate proxies are required.

To address this gap, this study proposes a computational framework for evaluating CBP measurement packages according to state observability, parameter identifiability, soft-sensor reconstruction performance, measurement burden, and robustness to practical measurement imperfections. A compact hybrid gray-box CBP model was used as a virtual plant to generate finite-difference output sensitivities, Fisher-information-based observability and identifiability metrics, parameter-correlation diagnostics, eigenvalue spectra, condition-number diagnostics, and approximate uncertainty measures. Rather than restricting the analysis to a small preselected list, the workflow evaluated all ethanol-mandatory combinations of the five modeled measurement channels: ethanol, soluble sugar, biomass proxy, enzyme-activity proxy, and residual-substrate proxy. These candidate sensor sets were then tested using a Monte Carlo unscented Kalman filter reconstruction experiment under model–plant mismatch and measurement noise conditions, with common plant-mismatch and initial-estimate realizations paired across sensor sets within each replicate. Additional analyses assessed the sensitivity of the sensor-set ranking to uniform and sensor-specific noise changes, alternative operating trajectories, missing measurements, assay delay, systematic measurement bias, alternative scoring weights, and alternative sensor-specific measurement-burden scenarios.

The contribution of this paper is threefold. First, it provides a pre-experimental computational pipeline for ranking CBP measurement candidates before wet-lab or pilot-plant implementation while making the candidate-set dependence of the ranking explicit. Second, it combines state observability, parameter identifiability, and nonlinear UKF reconstruction performance rather than relying only on endpoint prediction or product monitoring. Third, it evaluates whether the resulting ranking remains defensible under common measurement and implementation stress conditions, including sensor-specific noise, missingness, delay, bias, operating-trajectory variation, and measurement-burden assumptions. The results are intended to support digital-twin readiness assessment and experimental planning for CBP, not to claim experimental validation of a specific organism, sensor platform, or pilot-scale process.

## 2. Model, Sensor Sets, and Information-Based Analysis

### 2.1. Hybrid CBP Digital-Twin Model

A compact hybrid gray-box model serves as the computational digital-twin core for consolidated bioprocessing (CBP). The model was formulated to include the most significant dynamic couplings inherent to CBP, namely, growth of microorganism biomass, synthesis of cellulolytic enzymes, hydrolysis of insoluble substrate, accumulation and consumption of soluble sugars, and ethanol synthesis. Simplified mechanistic and hybrid models are common choices for bioprocess monitoring, state estimation, and control due to their interpretation potential and computationally-efficient structures [8,9,10]. In addition, modern bioprocess digital twins and hybrid models place particular emphasis on model compactness required for online state estimation and uncertainty analysis [11,19,20,33,34,35]. The state vector is defined as

(1) $$x\left(t\right)={\left[\begin{array}{ccccc} X(t) & E(t) & B(t) & C(t) & P(t) \end{array}\right]}^{⊤},$$

where *X* is biomass activity, *E* is enzymatic activity, *B* is residual insoluble substrate, *C* is soluble sugars concentration, and *P* is ethanol concentration. The operating variables included temperature and pH as

(2) $$u\left(t\right)={\left[\begin{array}{cc} T(t) & \mathrm{pH}(t) \end{array}\right]}^{⊤}.$$

The model describes CBP as three continuously varying regimes. The first regime represents growth and enzyme production, the second represents substrate hydrolysis, and the third represents ethanol fermentation. This phase-based structure corresponds to the well-known view of CBP as a process in which cellulase production, cellulose deconstruction, sugar release, and ethanol fermentation are coupled within a single operation [31,32]. Recent studies on CBP emphasize the role of this coupling in lignocellulosic conversion processes [5,6,7]. Related reduced-order CBP modeling and dynamic temperature–pH policy analyses have also shown that operating trajectories can strongly affect ethanol formation, conversion, productivity, and operating severity [36]. The phase weights are calculated using logistic functions as

(3) $$\begin{array}{cc} \phi_{1}\left(t\right) & =\;\frac{1}{1+\exp\{k\left(t-t_{1}\right)\}}, \end{array}$$

(4) $$\begin{array}{cc} \phi_{3}\left(t\right) & =\;\frac{1}{1+\exp\{-k\left(t-t_{2}\right)\}}, \end{array}$$

(5) $$\begin{array}{cc} \phi_{2}\left(t\right) & =\;\max\left(0,1-\phi_{1}\left(t\right)-\phi_{3}\left(t\right)\right), \end{array}$$

where k=0.28, $t_{1}=18$ h, and $t_{2}=44$ h. The weight functions were normalized so that their sum was equal to unity. This formulation does not cover all the regulatory mechanisms within the process as it was intended for use as a controlled dynamic benchmark in which the latent states were characterized by different time scales and levels of measurement relevance for observability and identifiability assessment.

The equations describing the nominal behavior of the system are written as   

(6) $$\begin{array}{cc} \frac{dX}{dt} & =\;\phi_{1}\left[\mu X\left(1-\frac{X}{K}\right)-dX\right]-0.0020\;\phi_{3}X, \end{array}$$

(7) $$\begin{array}{cc} \frac{dE}{dt} & =\;\phi_{1}\left[Y_{E}X-k_{\deg}E\right]-0.0040\;\phi_{3}E, \end{array}$$

(8) $$\begin{array}{cc} \frac{dB}{dt} & =\;-\phi_{2}v_{\mathrm{hyd}}, \end{array}$$

(9) $$\begin{array}{cc} \frac{dC}{dt} & =\;\phi_{2}\left(v_{\mathrm{hyd}}-0.10C\right)-\phi_{3}v_{\mathrm{ferm}}, \end{array}$$

(10) $$\begin{array}{cc} \frac{dP}{dt} & =\;\phi_{3}v_{\mathrm{ferm}}, \end{array}$$

where K=7.5 is the biomass carrying capacity constant. All growth, enzyme, hydrolysis, and fermentation dynamics depended on temperature and pH activity profiles. Hydrolysis and fermentation rates were computed as

(11) $$\begin{array}{cc} v_{\mathrm{hyd}} & =\;V_{\max}\left(\frac{B}{K_{m}+B+\epsilon }\right)\tanh\left(E\right), \end{array}$$

(12) $$\begin{array}{cc} v_{\mathrm{ferm}} & =\;Y_{P}\frac{C}{1+k_{\mathrm{inh}}P}, \end{array}$$

where ϵ is a small constant ensuring division by a non-zero number. Hydrolysis rate increased with residual substrate amount and enzymatic activity but was also limited by the tanh(E) factor. Fermentation rate transformed soluble sugar into ethanol with a feedback-dependent effect on product-inhibition dynamics. Despite its simplicity, the model preserved the core of the monitoring problem related to CBP: ethanol is a late product, and the reasons for poor batch performance could be traced in biomass, enzymes, substrate, and sugar states.

The nominal kinetic constants, activity functions, initial values, and numerical bounds used in the hybrid CBP virtual plant are summarized in Table 1.

Using this parameterization, the time-varying kinetic terms in Equations (6)–(12) were defined as

(13) $$\begin{array}{cc} \mu & =\;\mu_{0}f_{T,g}\left(T\right)f_{\mathrm{pH},g}\left(\mathrm{pH}\right)\theta_{\mu }, \end{array}$$

(14) $$\begin{array}{cc} Y_{E} & =\;Y_{E,0}f_{T,g}\left(T\right)f_{\mathrm{pH},g}\left(\mathrm{pH}\right)f_{\mathrm{pret}}\theta_{Y_{E}}, \end{array}$$

(15) $$\begin{array}{cc} V_{\max} & =\;V_{\max,0}f_{T,h}\left(T\right)f_{\mathrm{pH},h}\left(\mathrm{pH}\right)f_{\mathrm{pret}}\theta_{V_{\max}}, \end{array}$$

(16) $$\begin{array}{cc} Y_{P} & =\;Y_{P,0}f_{T,f}\left(T\right)f_{\mathrm{pH},f}\left(\mathrm{pH}\right)\theta_{Y_{P}}, \end{array}$$

(17) $$\begin{array}{cc} k_{\mathrm{inh}} & =\;k_{\mathrm{inh},0}\theta_{\mathrm{inh}}. \end{array}$$

The temperature and pH activity functions were defined as clipped Gaussian factors as

(18) $$\begin{array}{cc} f_{T,g}\left(T\right) & =\;\mathrm{clip}\left\{\exp\left[-{\left(\frac{T-48}{12.5}\right)}^{2}\right],0.15,1.25\right\}, \end{array}$$

(19) $$\begin{array}{cc} f_{T,h}\left(T\right) & =\;\mathrm{clip}\left\{\exp\left[-{\left(\frac{T-50}{13.0}\right)}^{2}\right],0.15,1.25\right\}, \end{array}$$

(20) $$\begin{array}{cc} f_{T,f}\left(T\right) & =\;\mathrm{clip}\left\{\exp\left[-{\left(\frac{T-42}{11.0}\right)}^{2}\right],0.12,1.20\right\}, \end{array}$$

(21) $$\begin{array}{cc} f_{\mathrm{pH},g}\left(\mathrm{pH}\right) & =\;\mathrm{clip}\left\{\exp\left[-{\left(\frac{\mathrm{pH}-5.6}{0.85}\right)}^{2}\right],0.15,1.25\right\}, \end{array}$$

(22) $$\begin{array}{cc} f_{\mathrm{pH},h}\left(\mathrm{pH}\right) & =\;\mathrm{clip}\left\{\exp\left[-{\left(\frac{\mathrm{pH}-5.2}{0.75}\right)}^{2}\right],0.15,1.25\right\}, \end{array}$$

(23) $$\begin{array}{cc} f_{\mathrm{pH},f}\left(\mathrm{pH}\right) & =\;\mathrm{clip}\left\{\exp\left[-{\left(\frac{\mathrm{pH}-6.0}{0.90}\right)}^{2}\right],0.15,1.20\right\}. \end{array}$$

Seven log-multiplicative uncertainty factors were used to facilitate the parameter identifiability analysis as

(24) $$\theta ={\left[\begin{array}{ccccccc} \theta_{\mu } & \theta_{Y_{E}} & \theta_{V_{\max}} & \theta_{Y_{P}} & \theta_{d} & \theta_{\mathrm{inh}} & \theta_{\mathrm{feed}} \end{array}\right]}^{⊤}.$$

They scaled growth rate, enzyme yield coefficient, hydrolysis capacity, ethanol yield, decay rate, inhibition rate, and feedstock accessibility, respectively. The logarithmic parameter transformation was appropriate because the kinetic uncertainties were multiplicative and because simultaneous biochemical effects can create practical identifiability degeneracies [28,29].

The assumed initial condition was

(25) $$x_{0}={\left[\begin{array}{ccccc} 0.10 & 0 & B_{0} & 0 & 0 \end{array}\right]}^{⊤},\;B_{0}=\frac{S_{0}}{5},$$

where $S_{0}=100$ and thus $B_{0}=20$ in the present study. The batch cycle duration was 96 h, while numerical integration was done by means of the fourth-order Runge–Kutta method with a 2 h internal time step. In order to conduct the observability and identifiability analysis, the open-loop control input (T,pH) was scheduled in such a way that all growth/enzyme synthesis, hydrolysis, and fermentation modes were activated as

(26) $$\left(T,\mathrm{pH}\right)=\left\{\begin{array}{cc} (48\;{}^{\circ}C,\;5.60), & t<24\;h,\\ (50\;{}^{\circ}C,\;5.20), & 24\leq t<54\;h,\\ (42\;{}^{\circ}C,\;6.00), & t\geq 54\;h. \end{array}\right.$$

This profile was not designed for optimal production of ethanol. The objective was to create a sufficiently representative trajectory to compare potential sensor suites. Excitation is vital in this respect as information-theoretic and sensitivity measures of identifiability are trajectory-dependent [37,38]. In other words, the model can be considered an experimental template for sensor suite design but not as a fully developed organism-specific CBP model.

### 2.2. Candidate Sensor Sets and Measurement Assumptions

The sensor library includes commonly used process measurements and potential proxy measurements for latent CBP states. Five modeled measurement channels are considered as

(27) M={P,C,X,E,B},

where *P*, *C*, *X*, *E*, and *B* denote ethanol, soluble sugar, a biomass proxy, an enzyme-activity proxy, and a residual insoluble-substrate proxy, respectively. This choice reflects a common monitoring challenge in CBP: product concentration is comparatively straightforward to monitor, whereas biological, enzymatic, hydrolysis-related, and intermediate-substrate states are sparse, delayed, or difficult to measure online. Soft-sensing approaches and process analytical technologies have been proposed to address similar monitoring limitations in bioprocess development [8,9,10,11,19,24].

The nominal measurement standard deviations and relative measurement-burden indices are summarized in Table 2. The burden index is dimensionless and represents relative sampling effort, assay latency, calibration burden, and online implementation difficulty rather than direct monetary cost. The nominal workflow assumes that ethanol and soluble sugar can be measured through routine at-line product and sugar analytics or calibrated spectroscopic/biosensor routes; biomass can be approximated using an optical, dielectric, or dry-weight-calibrated proxy; enzyme activity usually requires a higher-burden at-line or offline assay; and residual insoluble substrate requires a solids-related proxy or offline/at-line solids measurement. These values are therefore computational design assumptions for pre-experimental sensor-set comparison, not platform-calibrated constants. Sensitivity to alternative sensor-specific burden assumptions is examined later in the robustness analysis.

Ethanol is treated as the mandatory baseline measurement because it is the most direct product signal and represents the minimum product-monitoring configuration against which additional measurements are compared. To avoid limiting the recommendation to a small preselected candidate list, the analysis evaluates all ethanol-mandatory combinations formed by adding any subset of the remaining four measurement channels as

(28) $$S_{P}=\left\{\{P\}\cup A\;:\;A\subseteq \{C,X,E,B\}\right\}.$$

This formulation gives $2^{4}=16$ candidate sensor packages. The full candidate set is defined as

(29) $$\begin{array}{cccccccc} S_{1} & =\{P\}, & S_{2} & =\{P,C\}, & S_{3} & =\{P,X\}, & S_{4} & =\{P,E\},\\ S_{5} & =\{P,B\}, & S_{6} & =\{P,C,X\}, & S_{7} & =\{P,C,E\}, & S_{8} & =\{P,C,B\},\\ S_{9} & =\{P,X,E\}, & S_{10} & =\{P,X,B\}, & S_{11} & =\{P,E,B\}, & S_{12} & =\{P,C,X,E\},\\ S_{13} & =\{P,C,X,B\}, & S_{14} & =\{P,C,E,B\}, & S_{15} & =\{P,X,E,B\}, & S_{16} & =\{P,C,X,E,B\}. \end{array}$$

The ethanol-only set serves as the baseline because it represents the most straightforward product-monitoring configuration. The two- and three-channel sets test whether a small number of biochemical, biological, enzymatic, or hydrolysis-related measurements can substantially improve observability, identifiability, and state reconstruction. The four-channel sets test whether near-complete monitoring can provide most of the value of the full-proxy package while omitting one high-burden measurement. The full-proxy set represents the upper information benchmark within the modeled sensor library. Recent advances in soft sensing and state estimation in lignocellulosic and fermentation processes suggest that such measurement-set comparisons are necessary because hybrid sensors and state estimators depend strongly on which state variables are available for correction [39,40,41].

Measurement sensitivities are computed for sampling periods of 6, 12, and 24h. These periods represent increasingly sparse sampling scenarios in laboratory and pilot-scale operation. For a given sensor set S and sampling period $\Delta t_{s}$, the model outputs are obtained by concatenating the measurements at each sample point into a single vector as

(30) $$y_{S}={\left[\begin{array}{cccc} h_{S}{\left(x\left(t_{0}\right)\right)}^{⊤} & h_{S}{\left(x\left(t_{1}\right)\right)}^{⊤} & \cdots & h_{S}{\left(x\left(t_{N}\right)\right)}^{⊤} \end{array}\right]}^{⊤},$$

where $h_{S}\left(\cdot \right)$ selects the state components corresponding to the sensors in S. Each output component is weighted by its corresponding nominal measurement standard deviation, so that more precise measurements contribute more strongly to the weighted sensitivity and Fisher-information calculations.

The modeled measurement channels represent idealized scalar outputs that could be obtained using different online, at-line, or offline measurement technologies. Examples of practical process analytical technology (PAT), assay, and soft-sensor routes are summarized in Table 3. This distinction is important because a single process variable can be measured with different levels of delay, calibration burden, matrix sensitivity, drift behavior, detection-limit constraint, and online feasibility [39,40,41,42].

### 2.3. State Observability Analysis

State observability is assessed by evaluating the sensitivity of the weighted output vector to perturbations in the initial state. Given a sensor set S, the finite-difference sensitivity with respect to the *j*th initial state is computed from perturbed initial conditions as

(31) $$S_{x,j}=\frac{y_{S}\left(x_{0}+h_{j}^{+}e_{j}\right)-y_{S}\left(x_{0}-h_{j}^{-}e_{j}\right)}{h_{j}^{+}+h_{j}^{-}},$$

where $e_{j}$ is the *j*th unit vector. The nominal perturbation magnitude is defined as

(32) $$\varepsilon_{j}=10^{-3}\max(|x_{0,j}\left|,1\right)+10^{-4}.$$

For states whose negative perturbation does not violate nonnegativity, $h_{j}^{+}=h_{j}^{-}=\varepsilon_{j}$, which gives the usual symmetric finite difference. When the negative perturbation would produce a negative initial state, the lower perturbed value is clipped to the feasible nonnegative bound and the actual perturbation distance is used in the denominator. Thus,   

(33) $$h_{j}^{+}=\varepsilon_{j},\;h_{j}^{-}=x_{0,j}-\max\left(0,x_{0,j}-\varepsilon_{j}\right).$$

This definition avoids retaining a denominator of $2\varepsilon_{j}$ when the negative perturbation is clipped. For example, when $x_{0,j}=0$, the calculation becomes a one-sided finite difference with denominator $\varepsilon_{j}$. The actual perturbation distances used in the computation are recorded as part of the reproducibility output.

The weighted state-sensitivity matrix is then calculated as

(34) $${\tilde{S}}_{x}=W_{S}^{-1}S_{x},$$

where $W_{S}$ is the diagonal matrix of measurement standard deviations repeated over all sampling times. More precise measurements therefore receive larger weights in the information calculation. The state-information matrix is approximated as

(35) $$F_{x}={\tilde{S}}_{x}^{⊤}{\tilde{S}}_{x}.$$

This matrix is not the structural observability matrix in the differential geometric sense. Rather, it is a finite-horizon Fisher-information approximation that measures how much information the sampled outputs contain about perturbations in the initial states. This type of sensitivity-based information analysis is widely used for practical observability assessment in complex biological and nonlinear dynamical systems [29,37,38].

Let $\lambda_{i}\left(F_{x}\right)$ denote the eigenvalues of $F_{x}$. The numerical eigenvalue threshold is defined explicitly as

(36) $$\tau_{x}=\max\left(10^{-8}\lambda_{\max}\left(F_{x}\right),\;10^{-12}\right).$$

The numerical rank is then computed as

(37) $$r_{x}=\#\left\{i:\lambda_{i}\left(F_{x}\right)>\tau_{x}\right\}.$$

The log-pseudodeterminant is calculated over the active eigenvalues as

(38) $$\log_{10}\mathrm{pdet}\left(F_{x}\right)=\sum_{\lambda_{i}\left(F_{x}\right)>\tau_{x}}\log_{10}\left(\lambda_{i}\left(F_{x}\right)\right).$$

The minimum active eigenvalue is defined as

(39) $$\lambda_{\min,x}^{+}=\min\left\{\lambda_{i}\left(F_{x}\right):\lambda_{i}\left(F_{x}\right)>\tau_{x}\right\},$$

and the corresponding condition number is defined as

(40) $$\kappa \left(F_{x}\right)=\frac{\lambda_{\max}\left(F_{x}\right)}{\lambda_{\min,x}^{+}}.$$

If no eigenvalue exceeds the numerical threshold, the rank is set to zero, and the log-pseudodeterminant and condition-number diagnostics are treated as undefined or non-informative.

Approximate state uncertainty is estimated using a ridge-regularized pseudoinverse as

(41) $$\Sigma_{x}\approx {\left(F_{x}+\rho I\right)}^{†},\;\rho =10^{-10}.$$

Using these criteria, sensor ensembles can be compared not only by the total amount of information they provide but also by whether weakly informed state directions remain unresolved.

### 2.4. Parameter Identifiability Analysis

Parameter identifiability is analyzed using the same information-based framework, but with sensitivities computed with respect to logarithmically scaled model parameters instead of initial states. The parameter vector is defined as

(42) $$\theta ={\left[\begin{array}{ccccccc} \theta_{\mu } & \theta_{Y_{E}} & \theta_{V_{\max}} & \theta_{Y_{P}} & \theta_{d} & \theta_{\mathrm{inh}} & \theta_{\mathrm{feed}} \end{array}\right]}^{⊤},$$

where all nominal log-parameters are zero, corresponding to multiplicative scale factors of one. The finite-difference derivative of the output vector with respect to the *j*th log-parameter is computed as

(43) $$S_{\theta ,j}=\frac{y_{S}\left(\exp(\theta +\delta e_{j})\right)-y_{S}\left(\exp(\theta -\delta e_{j})\right)}{2\delta },\;\delta =10^{-3}.$$

The exponential mapping ensures that the perturbations correspond to relative changes in the kinetic and feedstock-accessibility factors. The weighted parameter-sensitivity matrix is then calculated as

(44) $${\tilde{S}}_{\theta }=W_{S}^{-1}S_{\theta },$$

and the corresponding Fisher-information approximation is

(45) $$F_{\theta }={\tilde{S}}_{\theta }^{⊤}{\tilde{S}}_{\theta }.$$

The same active-eigenvalue diagnostics used for state observability are computed for parameter identifiability. Specifically, the numerical threshold for the parameter-information matrix is defined as

(46) $$\tau_{\theta }=\max\left(10^{-8}\lambda_{\max}\left(F_{\theta }\right),\;10^{-12}\right),$$

the numerical rank is computed as

(47) $$r_{\theta }=\#\left\{i:\lambda_{i}\left(F_{\theta }\right)>\tau_{\theta }\right\},$$

and the active log-pseudodeterminant is calculated as

(48) $$\log_{10}\mathrm{pdet}\left(F_{\theta }\right)=\sum_{\lambda_{i}\left(F_{\theta }\right)>\tau_{\theta }}\log_{10}\left(\lambda_{i}\left(F_{\theta }\right)\right).$$

The minimum active eigenvalue and condition number are computed from the same active-eigenvalue set. These diagnostics report how much information is available in the identifiable parameter directions and whether some parameter directions remain weakly informed.

Because the active log-pseudodeterminant can change when the numerical rank changes, a fixed-dimension regularized determinant is also computed for the seven-dimensional parameter vector as

(49) $$D_{\theta ,\mathrm{reg}}=\log_{10}\det\left(F_{\theta }+\rho I_{7}\right)=\sum_{i=1}^{7}\log_{10}\left(\lambda_{i}\left(F_{\theta }\right)+\rho \right),\;\rho =10^{-10}.$$

This fixed-dimension metric provides a complementary D-optimality-style comparison that does not discard low-information parameter directions. The reported identifiability results therefore distinguish between active information volume, numerical-rank coverage, minimum eigenvalue behavior, and fixed-dimension regularized information.

Approximate parameter uncertainty is derived from the same ridge-regularized pseudoinverse as

(50) $$\Sigma_{\theta }\approx {\left(F_{\theta }+\rho I_{7}\right)}^{†},\;\rho =10^{-10},$$

with the standard error of the *j*th log-parameter estimated by

(51) $$\mathrm{SE}\left(\theta_{j}\right)=\sqrt{{\left[\Sigma_{\theta }\right]}_{jj}}.$$

The associated approximate multiplicative 95% uncertainty factor is defined as

(52) $$\exp\left(1.96\;\mathrm{SE}(\theta_{j})\right).$$

Correlations between parameters are defined as

(53) $$R_{ij}=\frac{{\left[\Sigma_{\theta }\right]}_{ij}}{\sqrt{{\left[\Sigma_{\theta }\right]}_{ii}{\left[\Sigma_{\theta }\right]}_{jj}}}.$$

Large off-diagonal correlation factors indicate parameter pairs that are difficult to estimate separately using the proposed measurements. This issue is important for partially observed bioprocesses because alternative kinetic parameter combinations can generate nearly identical ethanol or sugar time courses. When data are sparse, noisy, or insufficiently diverse, identifiability analysis provides a computational filter for selecting CBP measurement packages with stronger potential for useful parameter inference [28,29].

## 3. Soft-Sensor Evaluation, Sensor Ranking, and Robustness Assessment

### 3.1. Soft-Sensor Reconstruction Test

After the observability and identifiability analyses, each candidate sensor set was evaluated for nonlinear soft-sensing reconstruction under model–plant mismatch, initial-state uncertainty, and measurement noise conditions. An unscented Kalman filter (UKF) was used because it propagates mean and covariance information through nonlinear and phase-dependent dynamics without local linearization, which is important when some CBP states are only partially measured. Recent bioprocess monitoring studies emphasize that soft sensors, hybrid models, and model-based state-estimation methods are essential for digital bioprocessing because key physiological states are often unavailable from direct online measurements [11,40,41,43]. Related work on sensor-assisted bioprocess monitoring has also demonstrated the value of metabolic-heat-based soft sensing, spectroscopic monitoring, biomass estimation, soft-sensor recalibration, and joint state–parameter estimation for improving process-state reconstruction [12,13,14,15,16,17,18].

For the five-state CBP model, the UKF state estimate and covariance matrix at time $t_{k}$ are denoted by ${\hat{x}}_{k|k}$ and $P_{k|k}$, respectively. The sigma points were generated as

(54) $$\begin{array}{cc} \chi_{k|k}^{\left(0\right)} & =\;{\hat{x}}_{k|k}, \end{array}$$

(55) $$\begin{array}{cc} \chi_{k|k}^{\left(i\right)} & =\;{\hat{x}}_{k|k}+{\left[\sqrt{\left(n+\lambda \right)P_{k|k}}\right]}_{i},\;i=1,\ldots ,n, \end{array}$$

(56) $$\begin{array}{cc} \chi_{k|k}^{\left(i+n\right)} & =\;{\hat{x}}_{k|k}-{\left[\sqrt{\left(n+\lambda \right)P_{k|k}}\right]}_{i},\;i=1,\ldots ,n, \end{array}$$

with n=5 and

(57) $$\lambda =\alpha^{2}\left(n+\kappa \right)-n.$$

The UKF parameters were α=0.35, β=2, and κ=0. Each sigma point was propagated over one plant step using the same fourth-order Runge–Kutta integration scheme as the virtual plant:

(58) $$\chi_{k+1|k}^{\left(i\right)}=f_{\Delta t}\left(\chi_{k|k}^{\left(i\right)},u_{k},\theta_{\mathrm{model}}\right),$$

where $f_{\Delta t}\left(\cdot \right)$ denotes the CBP model integrated over one internal time step. The predicted mean and covariance were calculated as

(59) $$\begin{array}{cc} {\hat{x}}_{k+1|k} & =\;\sum_{i=0}^{2n}W_{i}^{\left(m\right)}\chi_{k+1|k}^{\left(i\right)}, \end{array}$$

(60) $$\begin{array}{cc} P_{k+1|k} & =\;Q+\sum_{i=0}^{2n}W_{i}^{\left(c\right)}\left(\chi_{k+1|k}^{\left(i\right)}-{\hat{x}}_{k+1|k}\right){\left(\chi_{k+1|k}^{\left(i\right)}-{\hat{x}}_{k+1|k}\right)}^{⊤}, \end{array}$$

where *Q* is the process-noise covariance matrix, and $W_{i}^{\left(m\right)}$ and $W_{i}^{\left(c\right)}$ are the conventional UKF mean and covariance weights.

At sampling instants, the measurement equation for sensor set S was

(61) $$y_{k}=h_{S}\left(x_{k}\right)+v_{k},\;v_{k}∼N\left(0,R_{S}\right),$$

where $R_{S}$ is the diagonal measurement-noise covariance matrix for the channels included in S. The predicted measurement sigma points and predicted measurement mean were calculated as

(62) $$\begin{array}{cc} z_{k+1|k}^{\left(i\right)} & =\;h_{S}\left(\chi_{k+1|k}^{\left(i\right)}\right), \end{array}$$

(63) $$\begin{array}{cc} {\hat{y}}_{k+1|k} & =\;\sum_{i=0}^{2n}W_{i}^{\left(m\right)}z_{k+1|k}^{\left(i\right)}. \end{array}$$

The innovation covariance matrix and state–measurement cross-covariance matrix were calculated as

(64) $$\begin{array}{cc} S_{k+1} & =\;R_{S}+\sum_{i=0}^{2n}W_{i}^{\left(c\right)}\left(z_{k+1|k}^{\left(i\right)}-{\hat{y}}_{k+1|k}\right){\left(z_{k+1|k}^{\left(i\right)}-{\hat{y}}_{k+1|k}\right)}^{⊤}, \end{array}$$

(65) $$\begin{array}{cc} C_{xy,k+1} & =\;\sum_{i=0}^{2n}W_{i}^{\left(c\right)}\left(\chi_{k+1|k}^{\left(i\right)}-{\hat{x}}_{k+1|k}\right){\left(z_{k+1|k}^{\left(i\right)}-{\hat{y}}_{k+1|k}\right)}^{⊤}. \end{array}$$

The Kalman gain and measurement-update step were computed as

(66) $$\begin{array}{cc} K_{k+1} & =\;C_{xy,k+1}S_{k+1}^{†}, \end{array}$$

(67) $$\begin{array}{cc} {\hat{x}}_{k+1|k+1} & =\;{\hat{x}}_{k+1|k}+K_{k+1}\left(y_{k+1}-{\hat{y}}_{k+1|k}\right), \end{array}$$

(68) $$\begin{array}{cc} P_{k+1|k+1} & =\;P_{k+1|k}-K_{k+1}S_{k+1}K_{k+1}^{⊤}. \end{array}$$

The pseudoinverse was used to improve numerical stability when innovation covariance matrices were close to singular.

Estimation accuracy was evaluated using a Monte Carlo simulation experiment. For each of the 16 ethanol-mandatory sensor-set configurations, $N_{\mathrm{MC}}=100$ simulation replicates were performed. In each replicate, multiplicative plant–model mismatch was imposed on the growth, enzyme-yield, hydrolysis-capacity, ethanol-yield, decay, inhibition, and feedstock-accessibility factors. The UKF uses the nominal model and therefore does not have access to the replicate-specific plant perturbation. Initial-state uncertainty was introduced by perturbing the initial state used by the estimator relative to the true plant initial state while enforcing physical nonnegativity bounds.

To ensure a fair paired comparison among sensor sets, the Monte Carlo design uses common plant and estimator realizations across all sensor packages. For a given replicate *r*, the same plant-parameter mismatch and the same initial-estimate perturbation were used for every candidate sensor set. Thus, differences in reconstruction error between two sensor sets are attributable to the measurement package rather than to different simulated plants. Measurement noise was generated consistently by channel: for each replicate, time point, and measurement channel, a channel-specific random-noise stream was used, so sensor sets sharing a channel received the same noise realization for that channel. Additional sensors introduced additional channel-specific noise streams without changing the underlying plant or initial-condition realization. This common-random-number design provides paired replicate-wise RMSE differences for the statistical comparisons.

For replicate *r*, the reconstruction error of state *j* is defined as

(69) $${\mathrm{RMSE}}_{j,r}=\sqrt{\frac{1}{N_{t}}\sum_{k=1}^{N_{t}}{\left({\hat{x}}_{j,k}^{\left(r\right)}-x_{j,k}^{\left(r\right)}\right)}^{2}},$$

where $N_{t}$ is the number of simulated time points. The latent-state RMSE was computed across the four non-product states:

(70) $${\mathrm{RMSE}}_{\mathrm{latent},r}=\frac{1}{4}\left({\mathrm{RMSE}}_{X,r}+{\mathrm{RMSE}}_{E,r}+{\mathrm{RMSE}}_{B,r}+{\mathrm{RMSE}}_{C,r}\right).$$

Ethanol is excluded from this latent-state average because ethanol is measured in every ethanol-mandatory candidate set and therefore does not represent a hidden-state reconstruction challenge. Additional reported statistics include the final absolute estimation error:

(71) $$e_{j,r}^{\mathrm{final}}=\left|{\hat{x}}_{j,N_{t}}^{\left(r\right)}-x_{j,N_{t}}^{\left(r\right)}\right|,$$

the mean absolute error, and the final covariance trace:

(72) $$\mathrm{tr}\left(P_{N_{t}|N_{t}}^{\left(r\right)}\right).$$

Each non-baseline sensor set was compared with ethanol-only monitoring using the paired replicate-specific latent-state RMSE differences:   

(73) $$d_{r}={\mathrm{RMSE}}_{\mathrm{latent},r}^{\mathrm{Ethanol}\;\mathrm{only}}-{\mathrm{RMSE}}_{\mathrm{latent},r}^{S}.$$

A positive value of $d_{r}$ indicates that sensor set S reduces latent-state reconstruction error relative to ethanol-only monitoring for the same replicate. Because the candidate space contains 16 ethanol-mandatory packages, there are 15 ethanol-only contrasts. The paired Wilcoxon signed-rank test was used as a secondary nonparametric comparison [44]. Bootstrap confidence intervals were also computed for the paired RMSE reduction, and false-discovery-rate-adjusted *p*-values are reported to account for the multiple ethanol-only contrasts. These statistical tests are not used to define the sensor ranking; instead, ranking is based on the combined observability, identifiability, UKF reconstruction, and measurement-burden scoring framework described below.

### 3.2. Scoring Sensor Values and Rankings

The final ranking step combines four criteria: state observability, parameter identifiability, UKF reconstruction accuracy, and measurement burden. Because these quantities have different units and numerical ranges, each metric is normalized to the interval [0,1] across the evaluated ethanol-mandatory candidate set. For a metric $m_{i}$ for which larger values are desirable, the normalized score is defined as

(74) $$\eta_{i}^{+}=\frac{m_{i}-\min_{j}\left(m_{j}\right)}{\max_{j}\left(m_{j}\right)-\min_{j}\left(m_{j}\right)}.$$

For a metric for which smaller values are desirable, the normalized score is defined as

(75) $$\eta_{i}^{-}=1-\frac{m_{i}-\min_{j}\left(m_{j}\right)}{\max_{j}\left(m_{j}\right)-\min_{j}\left(m_{j}\right)}.$$

If the metric range is zero, all normalized scores for that metric are set to 0.5. This min–max normalization places information value, reconstruction accuracy, and measurement burden on a common scale. However, because min–max normalization is candidate-list-dependent, the ranking should be interpreted as a ranking within the explicitly evaluated candidate space rather than as an absolute sensor value. The primary analysis therefore evaluated all 16 ethanol-mandatory combinations, and additional candidate-list, Pareto, and measurement-burden sensitivity outputs were generated to assess whether the recommendation depends on the set of candidates included in the comparison.

To score state observability, both total information volume and the weakest resolved active state direction were used:

(76) $$S_{\mathrm{obs}}=0.60\;\eta^{+}\left(\log_{10}\mathrm{pdet}\left(F_{x}\right)\right)+0.40\;\eta^{+}\left(\log_{10}\lambda_{\min,x}^{+}\right),$$

where $\lambda_{\min,x}^{+}$ is the minimum eigenvalue of $F_{x}$ above the numerical threshold defined in Section 2.3. Parameter identifiability is scored in the same way:

(77) $$S_{\mathrm{id}}=0.60\;\eta^{+}\left(\log_{10}\mathrm{pdet}\left(F_{\theta }\right)\right)+0.40\;\eta^{+}\left(\log_{10}\lambda_{\min,\theta }^{+}\right).$$

The pseudodeterminant term rewards total active information volume, whereas the minimum-eigenvalue term penalizes sensor sets that leave at least one active state or parameter direction weakly informed. This avoids assigning a high score to a configuration that performs well only in a small number of dominant directions.

The UKF reconstruction score is derived from the Monte Carlo mean latent-state RMSE:

(78) $$S_{\mathrm{ukf}}=\eta^{-}\left({\bar{\mathrm{RMSE}}}_{\mathrm{latent}}\right),$$

where ${\bar{\mathrm{RMSE}}}_{\mathrm{latent}}$ is the mean latent-state RMSE across the 100 paired Monte Carlo replicates. The measurement-burden score is defined as

(79) $$S_{\mathrm{burden}}=\eta^{-}\left(c_{S}\right),$$

where $c_{S}$ is the total dimensionless measurement-burden index of sensor set S.

The primary aggregate sensor-value score is then calculated as

(80) $$S_{\mathrm{total}}=0.30S_{\mathrm{obs}}+0.35S_{\mathrm{id}}+0.25S_{\mathrm{ukf}}+0.10S_{\mathrm{burden}}.$$

The identifiability term is assigned a slightly larger weight than the observability term because the envisioned digital-twin use case includes both state reconstruction and model learning through parameter refinement. The burden term is included to discourage automatically selecting the most measurement-intensive package when a reduced package gives comparable information and reconstruction value.

A secondary value-per-burden diagnostic is also calculated as

(81) $$S_{\mathrm{value}/\mathrm{burden}}=\frac{S_{\mathrm{total}}}{c_{S}}.$$

This quantity is not used as the primary ranking criterion. Instead, it is used to interpret trade-offs between information gain and implementation burden. In addition, Pareto screening was performed using information, reconstruction accuracy, and measurement burden so that candidate packages can be identified as dominated or non-dominated. A sensor set is considered dominated if another evaluated package provides no worse information and reconstruction performance while having no greater burden. These diagnostics help distinguish the best aggregate package from lower-burden alternatives and from full-proxy monitoring, which represents the upper measurement-completeness benchmark.

### 3.3. Noise, Operating-Trajectory, and Scoring Robustness Analyses

Robustness analyses were performed to assess whether the sensor-set hierarchy depends strongly on measurement-quality assumptions, operating trajectory, measurement imperfections, scoring weights, or measurement-burden assumptions. This step is important because laboratories may differ in sensor calibration, assay availability, online implementation difficulty, and the relative priority placed on observability, identifiability, reconstruction accuracy, and burden.

First, a uniform measurement-noise sensitivity analysis was performed using three noise multipliers as

(82) $$\gamma_{\sigma }\in \left\{0.5,\;1.0,\;2.0\right\}.$$

In this analysis, all nominal sensor standard deviations are scaled as

(83) $$\sigma_{S,i}^{\left(\gamma \right)}=\gamma_{\sigma }\sigma_{S,i}.$$

The weighted sensitivity matrices, Fisher-information metrics, and aggregate scores were then recomputed. Because Fisher-information matrices depend on the inverse measurement variance, this analysis tested whether the ranking was preserved when all measurements were assumed to be uniformly more accurate or less accurate.

Second, an independent sensor-specific noise analysis was performed so that sensor channels could improve or degrade independently. For each scenario, the standard deviation of each sensor k∈{P,C,X,E,B} was multiplied by an independently sampled factor as

(84) $$\sigma_{k}^{\left(m\right)}=m_{k}\sigma_{k},\;m_{k}\in \left[0.5,\;2.0\right].$$

A total of 200 independent sensor-noise scenarios were evaluated. For each scenario, the observability and identifiability matrices were reweighted, the aggregate score was recomputed across the 16 ethanol-mandatory candidates, and the rank distribution was recorded. This analysis tested whether the ranking was stable when one sensor channel became noisier or more accurate relative to the others.

Third, the effect of operating trajectory was examined because sensitivity-based observability and identifiability metrics are trajectory-dependent. In addition to the nominal temperature–pH schedule, four alternative feasible schedules were tested: a milder excitation profile, an extended hydrolysis profile, an earlier fermentation profile, and a shifted feasible profile in which temperature–pH levels were moved away from the nominal values while keeping all process phases active. For each trajectory, the observability, identifiability, scoring, and ranking calculations were repeated for all candidate sensor sets and sampling intervals. The resulting rankings were compared with the nominal ranking using rank-correlation and maximum-rank-shift diagnostics.

Fourth, full UKF stress tests were performed for practical measurement imperfections. Unlike an information-only approximation, these tests reran the complete reconstruction workflow under stressed measurement conditions and then recomputed the corresponding ranking. Random missingness is represented by dropping measurement updates according to specified channel-availability probabilities. Two missingness cases were considered: 20% missing observations for all sensors and higher missingness for biomass, enzyme, and substrate proxy channels. Assay delay was tested by delaying measurement availability by 6h before UKF correction. Systematic measurement bias was injected directly as an additive offset in the affected measurement channels rather than being treated only as zero-mean variance. Two bias cases are considered: a moderate all-sensor bias of $0.5\sigma_{k}$ and a proxy-bias case in which biomass, enzyme, and substrate proxy channels carry larger bias than ethanol and sugar measurements.

Fifth, the aggregate score was recalculated using alternative weighting schemes. For each weighting scheme,

(85) $$w=\left[\begin{array}{cccc} w_{\mathrm{obs}} & w_{\mathrm{id}} & w_{\mathrm{ukf}} & w_{\mathrm{burden}} \end{array}\right],\;\sum_{i}w_{i}=1,$$

and

(86) $$S_{\mathrm{total}}^{\left(w\right)}=w_{\mathrm{obs}}S_{\mathrm{obs}}+w_{\mathrm{id}}S_{\mathrm{id}}+w_{\mathrm{ukf}}S_{\mathrm{ukf}}+w_{\mathrm{burden}}S_{\mathrm{burden}}.$$

The following weight vectors were evaluated as

(87) $$\begin{array}{cc} w_{\mathrm{primary}} & =\;\left[\begin{array}{cccc} 0.30 & 0.35 & 0.25 & 0.10 \end{array}\right], \end{array}$$

(88) $$\begin{array}{cc} w_{\mathrm{equal}} & =\;\left[\begin{array}{cccc} 0.25 & 0.25 & 0.25 & 0.25 \end{array}\right], \end{array}$$

(89) $$\begin{array}{cc} w_{\mathrm{obs}} & =\;\left[\begin{array}{cccc} 0.55 & 0.20 & 0.15 & 0.10 \end{array}\right], \end{array}$$

(90) $$\begin{array}{cc} w_{\mathrm{id}} & =\;\left[\begin{array}{cccc} 0.15 & 0.60 & 0.15 & 0.10 \end{array}\right], \end{array}$$

(91) $$\begin{array}{cc} w_{\mathrm{ukf}} & =\;\left[\begin{array}{cccc} 0.20 & 0.20 & 0.50 & 0.10 \end{array}\right], \end{array}$$

(92) $$\begin{array}{cc} w_{\mathrm{burden}-\mathrm{sensitive}} & =\;\left[\begin{array}{cccc} 0.25 & 0.30 & 0.20 & 0.25 \end{array}\right], \end{array}$$

(93) $$\begin{array}{cc} w_{\mathrm{burden}-\mathrm{averse}} & =\;\left[\begin{array}{cccc} 0.20 & 0.20 & 0.15 & 0.45 \end{array}\right]. \end{array}$$

These cases represent balanced performance, observation-oriented design, identification-oriented design, reconstruction-oriented design, burden-sensitive preference, and strongly burden-averse preference.

Finally, alternative sensor-specific measurement-burden scenarios were tested. These scenarios changed the channel-specific burden indices to represent different practical workflows, such as a spectroscopy-assisted workflow with lower burden for calibrated optical measurements, an offline-assay workflow with higher burden for enzyme and residual-substrate measurements, and a solids-intensive workflow with elevated burden for residual insoluble-substrate monitoring. The aggregate rankings were recomputed in each workflow-specific burden scenario. This analysis separated the effect of changing score weights from the effect of changing the assumed practical cost of individual sensor channels.

Together, these robustness analyses tested whether a sensor package remained attractive when measurement noise, operating policy, data missingness, assay delay, systematic bias, scoring priorities, and measurement-burden assumptions were varied. A package that maintains a high rank across these cases is more defensible for pre-experimental CBP digital-twin planning, whereas a package preferred only in one weighting or workflow scenario should be interpreted as objective specific rather than universally optimal.

**Figure 1:**
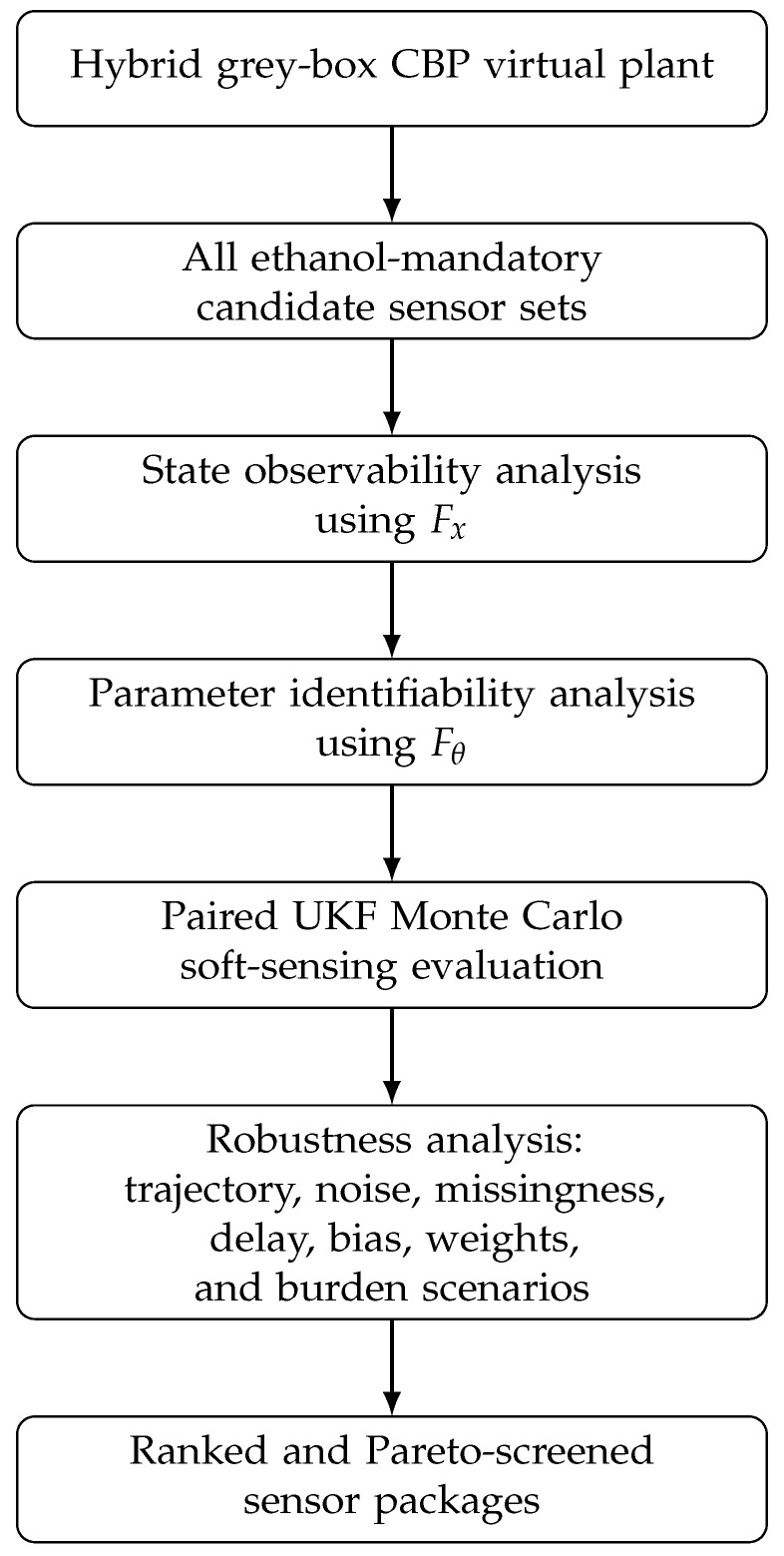
Workflow for the proposed sensor-set design framework for digital-twin-assisted consolidated bioprocessing. Starting from a hybrid gray-box CBP virtual plant, all ethanol-mandatory measurement packages are defined and evaluated through state-observability analysis, parameter-identifiability analysis, and paired UKF-based Monte Carlo soft-sensing reconstruction. The candidate packages are then examined using robustness analyses involving alternative operating trajectories, measurement-noise assumptions, missing-data scenarios, assay delay, systematic measurement bias, alternative scoring weights, and alternative sensor-specific measurement-burden scenarios. The workflow yields a ranked and Pareto-screened list of sensor packages for assessing CBP digital-twin readiness before experimental or pilot-scale implementation.

The workflow used for pre-ranking CBP sensor packages before detailed soft-sensor evaluation and digital-twin deployment is shown in Figure 1.

### 3.4. Computational Reproducibility

All simulations, state-estimation routines, sensitivity analyses, statistical comparisons, tables, and figures were implemented in Python 3.13.5. The final production run was executed with the base random seed 42. The production run used the ethanol-mandatory candidate-set mode, giving 16 candidate sensor packages, and used $N_{\mathrm{MC}}=100$ Monte Carlo replicates for each candidate sensor set.

The hybrid CBP virtual plant was simulated using a fixed-step fourth-order Runge–Kutta scheme. The same numerical integration approach was used for nominal trajectory simulation, finite-difference sensitivity analysis, and UKF prediction. The production run used GPU acceleration for vectorized finite-difference sensitivity batches when available, with automatic CPU fallback. The final run used an NVIDIA GeForce RTX 2070 for the GPU-enabled sensitivity calculations. The model equations, sensor definitions, observability and identifiability metrics, UKF update equations, scoring procedure, and robustness-test definitions are provided in Section 2 and Section 3. Additional UKF Monte Carlo settings required to reproduce the soft-sensing RMSE values and pairwise statistical comparisons are summarized in Table 4.

The numerical values in this study were treated as computational design assumptions for comparing candidate sensor packages, not as platform-calibrated experimental constants. The assumptions are consistent with the use of Fisher-information-based experimental design, nonlinear state estimation, and soft-sensing analysis in partially observed bioprocess systems [9,11,27,28,37,38]. The main assumptions are summarized in Table 5.

## 4. Results and Discussion

### 4.1. Nominal CBP Trajectory with the Excitation Schedule

**Figure 2:**
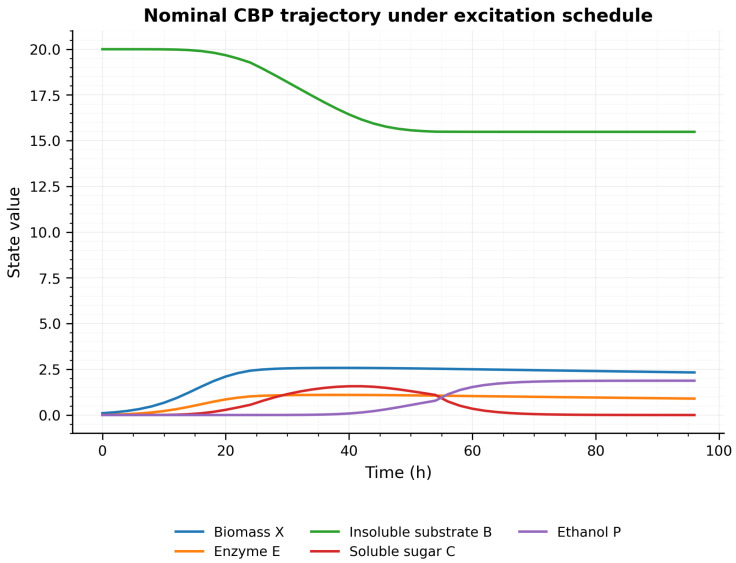
Nominal CBP trajectory with the temperature–pH excitation schedule, showing biomass growth, enzyme formation, residual insoluble-substrate conversion, soluble sugar accumulation, and ethanol formation.

The nominal CBP trajectory with the temperature–pH excitation profile showed the expected phase-dependent behavior. Biomass and enzyme activity increased mainly during the early phase, hydrolysis increased soluble sugar during the intermediate phase, and ethanol accumulation became dominant during the later phase, as shown in Figure 2. This behavior is consistent with CBP as a coupled process involving growth, enzyme production, substrate deconstruction, sugar release, and product formation on different time scales [6,7,31,32]. The delayed ethanol response also illustrates why ethanol-only monitoring is insufficient for diagnosing earlier causes of poor conversion, such as weak growth, insufficient enzyme activity, limited hydrolysis, or sugar limitation.

### 4.2. State-Observability Enhancement with Increasingly Informative Sensors

**Figure 3:**
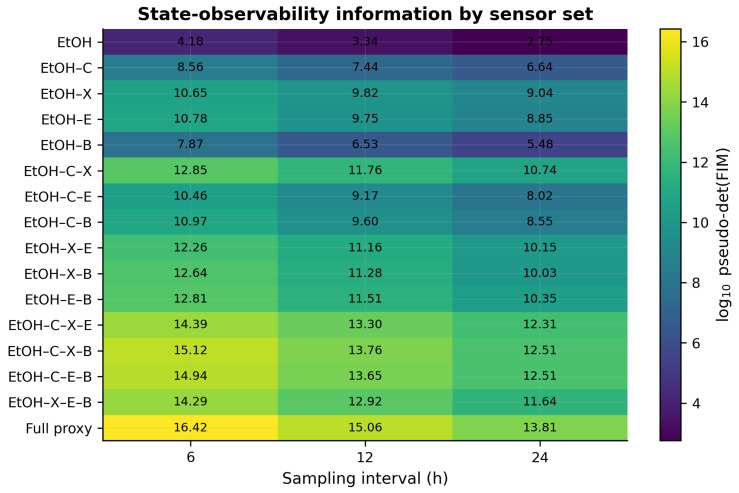
State-observability information for the 16 ethanol-mandatory sensor sets and sampling intervals, measured using the log-pseudodeterminant of the Fisher-information-type observability matrix.

State-observability information increased substantially when additional measurements were added to ethanol-only monitoring. Across the 16 ethanol-mandatory candidate packages, ethanol-only monitoring provided the lowest state-information content, as shown in Figure 3. At a 6h sampling interval, the state-observability log-pseudodeterminant increased from 4.18 with ethanol-only monitoring to 8.56 after soluble sugar was added. Full-proxy monitoring gave the largest state-observability information, with values of 16.42, 15.06, and 13.81 at sampling intervals of 6, 12, and 24h, respectively.

The all-combination analysis also identified strong reduced four-channel packages. The ethanol–sugar–biomass–substrate package reached state-observability log-pseudodeterminants of 15.12, 13.76, and 12.51 at 6, 12, and 24h, respectively, while the ethanol–sugar–enzyme–substrate package gave very similar values of 14.94, 13.65, and 12.51. These results show that residual-substrate information can provide a major state-observability gain when combined with ethanol, sugar, and a biological or enzymatic proxy. The previously emphasized ethanol–sugar–biomass–enzyme package also remained informative, with values of 14.39, 13.30, and 12.31, but it no longer represented the strongest reduced observability option once all ethanol-mandatory combinations were included.

Overall, the observability analysis confirms that product-only sensing is weak for state-aware CBP digital twins. Intermediate and latent-state proxy measurements provide much stronger information about the initial-state directions that drive the batch trajectory. This finding agrees with broader bioprocess-monitoring experience, where endpoint or product signals alone are often insufficient for reconstructing hidden physiological states, whereas intermediate measurements and proxy variables can substantially improve soft-sensor performance [8,9,10,11]. Within the tested candidate set, full-proxy monitoring remained the maximum state-observability benchmark, while ethanol–sugar–biomass–substrate and ethanol–sugar–enzyme–substrate provided strong reduced alternatives.

### 4.3. Parameter-Identifiability Improvement with Biomass and Enzyme Sensors

**Figure 4:**
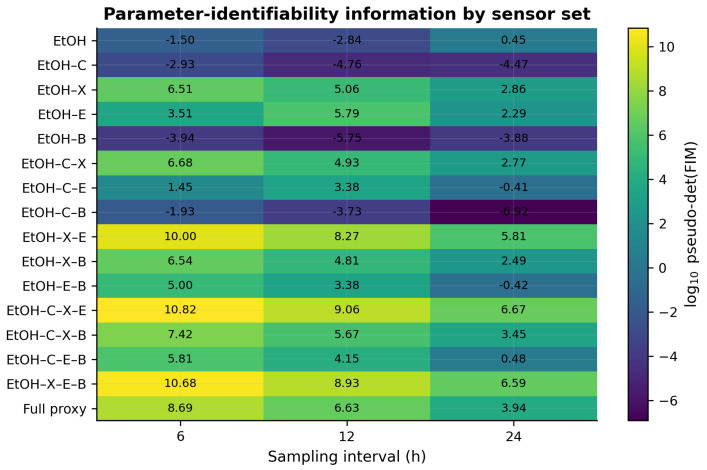
Parameter-identifiability information for the 16 ethanol-mandatory sensor sets and sampling intervals, measured using the active log-pseudodeterminant of the parameter Fisher information matrix.

The parameter-identifiability results were more nuanced than the state-observability results because different metrics emphasize different properties of the Fisher information matrix. For the active log-pseudodeterminant criterion, the ethanol–sugar–biomass–enzyme package gave the strongest parameter-identifiability performance among the evaluated packages, as shown in Figure 4. Its log-pseudodeterminants were 10.82, 9.06, and 6.67 at sampling intervals of 6, 12, and 24h, respectively. The ethanol–biomass–enzyme–substrate package was close behind, with values of 10.68, 8.93, and 6.59. These results show that biomass and enzyme proxies are especially informative for separating growth, enzyme-production, and hydrolysis-related parameter effects.

For comparison, full-proxy monitoring gave active parameter log-pseudodeterminants of 8.69, 6.63, and 3.94 at 6, 12, and 24h, respectively. The lower active pseudodeterminant for the full-proxy set does not mean that the full-proxy set is less informative in an absolute sense. Rather, it reflects the behavior of the active pseudodeterminant when additional weak eigenvalue directions are retained. The pseudodeterminant sums only eigenvalues above the numerical threshold, so it can favor a reduced package whose information is concentrated in fewer active directions.

**Figure 5:**
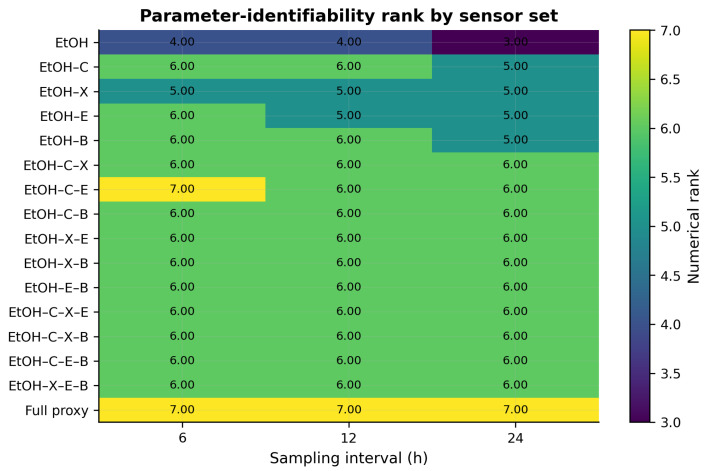
Numerical parameter-identifiability rank for the 16 ethanol-mandatory sensor sets and sampling intervals. Higher rank indicates that more independent parameter directions are practically resolved by the available measurements.

The numerical-rank results clarify this interpretation, as shown in Figure 5. Although the ethanol–sugar–biomass–enzyme package had the largest active-information volume, its parameter-information matrix had numerical rank 6 in the tested sampling cases. In contrast, full-proxy monitoring retained full numerical rank 7 across the tested sampling intervals. Therefore, the ethanol–sugar–biomass–enzyme package is best viewed as the strongest active-information package for parameter learning, whereas full-proxy monitoring provides the most complete coverage of all seven parameter directions.

**Figure 6:**
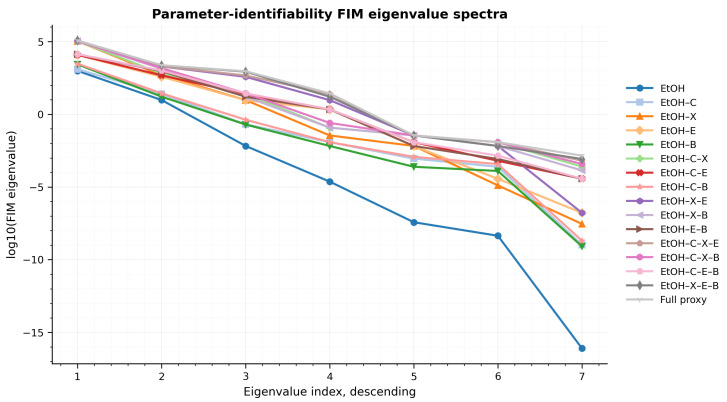
Eigenvalue spectra of selected parameter Fisher information matrices at the 12h sampling interval. Larger eigenvalues indicate stronger parameter-information directions, while very small eigenvalues indicate weakly resolved or practically non-identifiable parameter combinations.

The eigenvalue spectra further illustrate the distinction between active information volume and full-dimensional coverage, as shown in Figure 6. In the 12h case, the ethanol–sugar–biomass–enzyme package produced a larger active pseudodeterminant than full-proxy monitoring, but the full-proxy set preserved an additional weak parameter direction and gave the larger fixed-dimension regularized determinant. The fixed-dimension regularized log determinant was 6.94 for full-proxy monitoring and 6.36 for the ethanol–sugar–biomass–enzyme package. Thus, full-proxy monitoring remains the most complete parameter-information configuration, while ethanol–sugar–biomass–enzyme is the strongest reduced package for the active-information criterion.

The difference between observability and identifiability is therefore important. A sensor configuration that is strong for state reconstruction is not necessarily the same configuration that is most efficient for parameter identification. Identifiability depends on whether parameter perturbations produce sufficiently distinct output responses. In the present model, biomass and enzyme measurements complement ethanol and sugar measurements by helping to separate growth, enzyme-yield, and hydrolysis effects from ethanol-yield, inhibition, and feedstock-accessibility effects. Residual-substrate information, by contrast, was especially valuable for state observability and the aggregate ranking, but it did not replace the parameter-learning value of the enzyme proxy.

**Figure 7:**
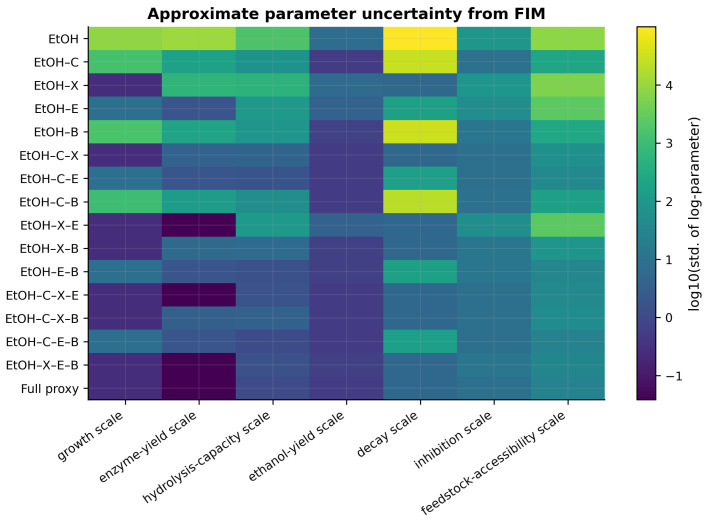
Approximate parameter uncertainty estimated from the inverse Fisher information matrix. Lower values indicate better parameter precision.

**Figure 8:**
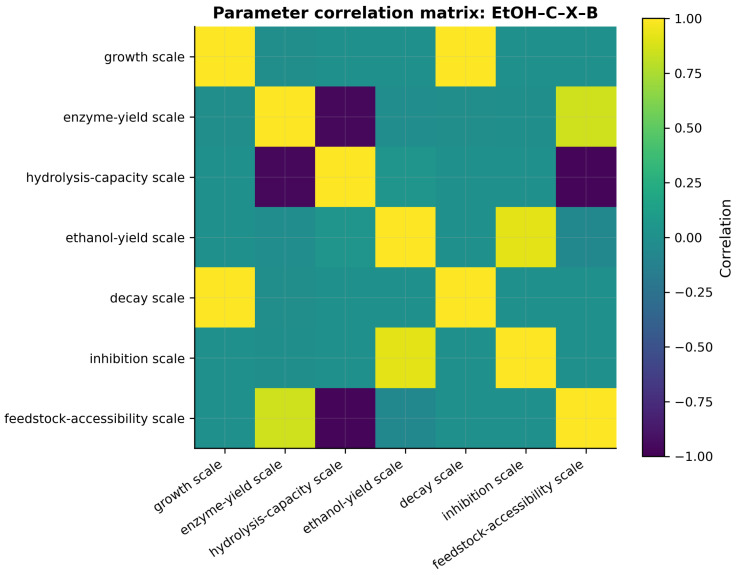
Parameter correlation matrix for the full-proxy monitoring case. Strong positive or negative off-diagonal values indicate parameter pairs that remain difficult to distinguish.

The inclusion of biomass and enzyme surrogates lowered the uncertainty of parameters associated with growth, enzyme production, and hydrolysis, as shown in Figure 7. However, strong correlations were still observed for selected parameter pairs, especially growth and decay, ethanol yield and inhibition, and hydrolysis capacity and feedstock accessibility, as shown in Figure 8. These correlations show that some biological mechanisms can still produce similar measured trajectories even when additional proxy measurements are available. Therefore, Fisher-information metrics, eigenvalue spectra, fixed-dimension regularized determinants, and parameter-correlation diagnostics should be used together when judging practical identifiability and selecting sensor packages before experimental implementation [28,37,38].

### 4.4. Impact of the Sensor Set on UKF Reconstruction Quality

More informative sensor sets improved latent-state reconstruction under model–plant mismatch conditions. Ethanol-only monitoring gave the weakest reconstruction performance, with a mean latent-state RMSE of 1.1899. Adding soluble sugar alone reduced the mean latent-state RMSE only slightly to 1.1398, confirming that a minimal product–sugar package is still insufficient for reconstructing hidden biomass, enzyme, and residual-substrate dynamics. In contrast, packages that included residual-substrate measurements strongly reduced substrate RMSE, while packages that included biomass and enzyme proxies improved the corresponding biological and enzymatic state estimates. These trends are consistent with the observability results and with previous bioprocess soft-sensing studies, where estimator performance depends strongly on whether the available measurements excite the dominant latent-state directions [8,9,10,11].

**Figure 9:**
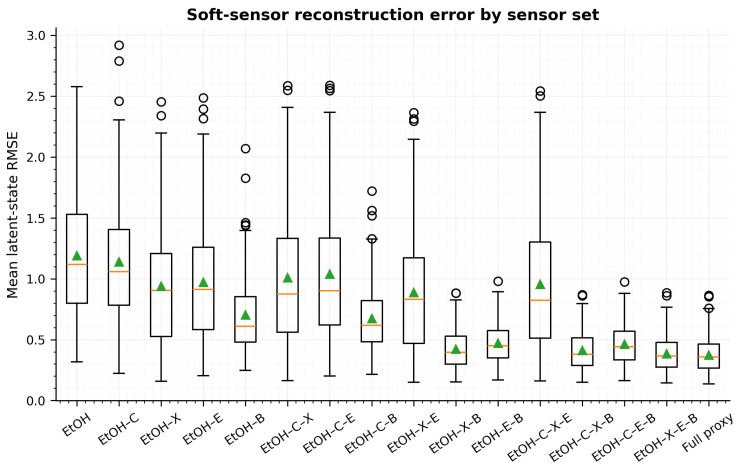
Monte Carlo distribution of mean UKF latent-state reconstruction RMSE by sensor set. Lower RMSE indicates better reconstruction of the unmeasured or partially measured CBP states across paired model–plant mismatch simulations.

Among all 16 ethanol-mandatory packages, full-proxy monitoring gave the lowest mean latent-state RMSE, 0.3756. The ethanol–biomass–enzyme–substrate package was close behind with 0.3843, followed by the top aggregate-scoring ethanol–sugar–biomass–substrate package with 0.4121. Thus, full-proxy monitoring remained the best reconstruction benchmark, but several four-channel packages achieved similar UKF accuracy with lower measurement burden. The Monte Carlo distributions of latent-state RMSE and representative state-wise errors are shown in Figure 9 and Table 6.

Paired Wilcoxon tests were used to compare each non-baseline sensor set with ethanol-only monitoring because the replicate-wise RMSE differences were not assumed to be normally distributed [44]. The revised Monte Carlo design used common plant mismatch and initial-estimate perturbations across all sensor sets, so the replicate-wise RMSE differences were paired observations. In addition to the raw test statistics, bootstrap confidence intervals were calculated for mean RMSE reductions, and the Wilcoxon *p*-values were adjusted across the 15 ethanol-only contrasts.

All non-baseline sensor sets reduced mean latent-state RMSE relative to ethanol-only monitoring. The largest reconstruction improvement was obtained with full-proxy monitoring, which reduced mean latent-state RMSE from 1.1899 to 0.3756. The corresponding mean reduction was 0.8143, with a bootstrap 95% interval of 0.7398–0.8894. The ethanol–biomass–enzyme–substrate package provided a nearly equivalent reconstruction reduction of 0.8056, while the ethanol–sugar–biomass–substrate package reduced RMSE by 0.7778. These results show that residual-substrate information is especially valuable for UKF state reconstruction when combined with biomass or enzyme proxies. The paired comparison results are summarized in Table 7.

### 4.5. Recommended Sensor-Set Ranking and Measurement-Burden Trade-Off

The aggregate sensor-value ranking changed when all 16 ethanol-mandatory combinations were evaluated instead of only the original seven preselected packages. The ethanol–sugar–biomass–substrate package achieved the highest overall score, followed by full-proxy monitoring and the ethanol–biomass–enzyme–substrate package, as shown in Table 8. Ethanol-only monitoring remained the least effective option, confirming that product-only sensing is inadequate when state observability, parameter identifiability, nonlinear reconstruction accuracy, and measurement burden are considered together.

This ordering clarifies the trade-off among information value, reconstruction accuracy, and measurement burden. Full-proxy monitoring gave the best UKF reconstruction accuracy and the most complete measurement coverage, but it also had the highest burden index. The ethanol–sugar–biomass–substrate package ranked first overall because it combined strong state observability, high UKF reconstruction accuracy, competitive identifiability, and lower burden than the full-proxy package. The ethanol–biomass–enzyme–substrate package gave nearly full-proxy reconstruction performance and a strong parameter-identifiability score, but its higher burden and lack of a sugar measurement placed it below the top-ranked ethanol–sugar–biomass–substrate package in the aggregate ranking.

The previously emphasized ethanol–sugar–biomass–enzyme package remained important for parameter learning and ranked sixth overall, but it was no longer the best reduced aggregate package once residual-substrate-containing combinations were included. Therefore, the practical recommendation is conditional on the design objective: full-proxy monitoring is preferred when maximum reconstruction and completeness are required; ethanol–sugar–biomass–substrate is preferred for the primary aggregate score; and ethanol–sugar–biomass–enzyme remains a strong reduced option when parameter identifiability is prioritized. This supports the broader digital bioprocessing view that process analytical measurements, state-estimation methods, and uncertainty-aware decision support should be integrated before closed-loop digital-twin deployment [11,19,20,24].

### 4.6. Robustness to Measurement Noise, Operating Trajectories, Measurement Imperfections, and Scoring Weights

**Figure 10:**
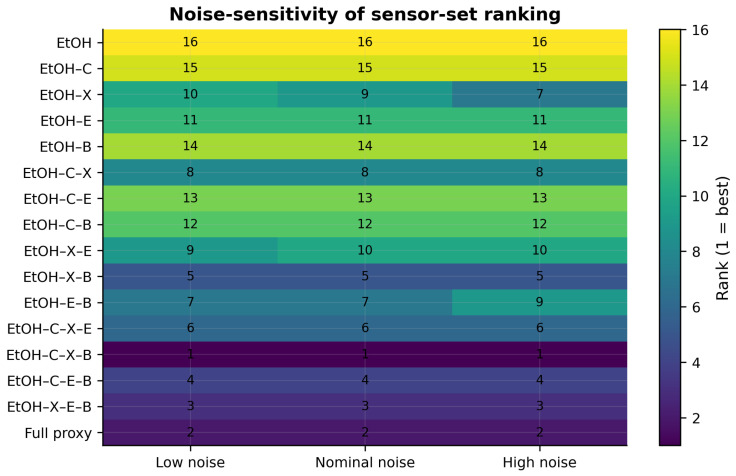
Sensitivity of sensor-set ranking to uniform measurement-noise scaling. Rank 1 denotes the best-performing sensor set under the corresponding noise assumption.

The robustness analyses showed that the main recommendation was generally stable with changes in measurement quality, operating trajectory, measurement imperfections, scoring weights, and sensor-specific burden assumptions. With uniform measurement-noise scaling, the ethanol–sugar–biomass–substrate package remained top-ranked in the low-noise, nominal-noise, and high-noise cases, as shown in Figure 10 and Table 9. Spearman rank correlations relative to the nominal-noise ranking were close to one, and the maximum rank shift was at most two. This indicates that the primary ranking was not an artifact of a single assumed global measurement-noise level. Such robustness is important because sensor quality, assay uncertainty, and data availability can differ substantially across laboratories and development stages [11,19,24].

**Figure 11:**
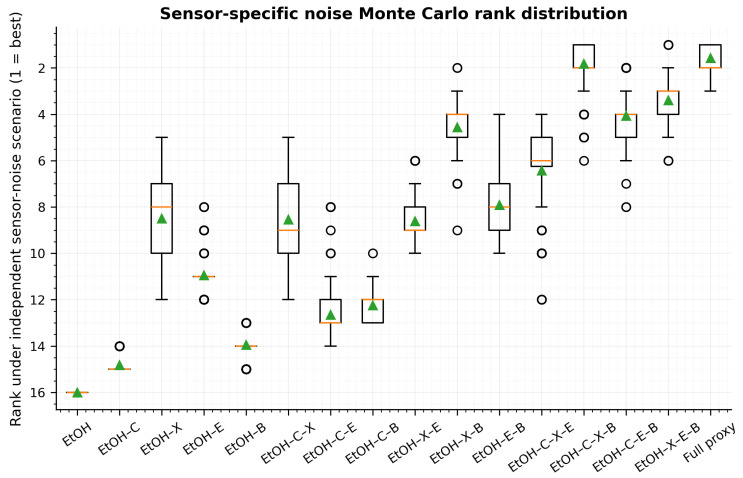
Rank distribution under 200 independent sensor-specific noise scenarios. Each sensor standard deviation was independently varied before recalculating the sensor-set scores.

When individual sensor-noise levels were varied independently, the competition between the top reduced package and full-proxy monitoring became clearer, as shown in Figure 11. Across 200 sensor-specific noise scenarios, full-proxy monitoring had the best mean rank, 1.57, and was ranked first in 99 scenarios. The ethanol–sugar–biomass–substrate package had a mean rank of 1.83 and was ranked first in 96 scenarios. The ethanol–biomass–enzyme–substrate package ranked first in five scenarios. Thus, the full-proxy package is slightly more robust when individual sensor-noise assumptions vary, whereas ethanol–sugar–biomass–substrate remains the strongest lower-burden aggregate recommendation.

**Figure 12:**
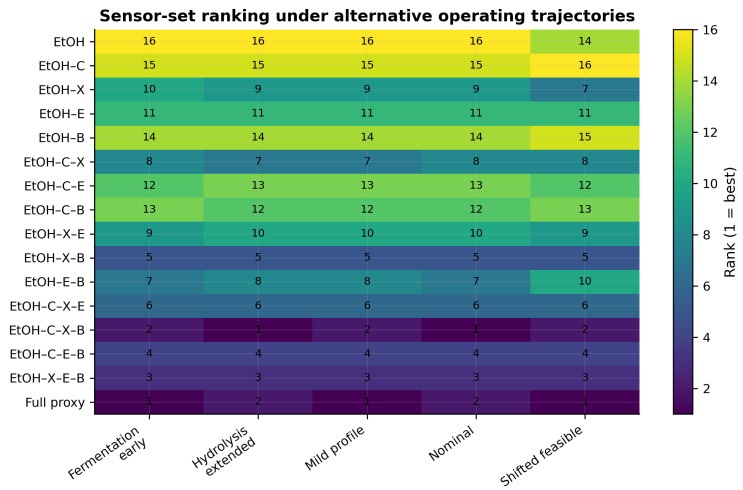
Sensor-set ranking for alternative feasible operating trajectories. Rank 1 denotes the highest aggregate sensor-value score for each temperature–pH trajectory.

The operating-trajectory analysis confirmed that the ranking was not driven only by the nominal temperature–pH schedule, as shown in Figure 12 and Table 10. Full-proxy monitoring provided the highest state-observability score for all tested trajectories. The top aggregate package was ethanol–sugar–biomass–substrate with the nominal and hydrolysis-extended trajectories, while full-proxy monitoring became the top aggregate package for the mild, fermentation-early, and shifted-feasible trajectories. The Spearman correlation relative to the nominal trajectory ranged from 0.9647 to 1.0000, with a maximum rank shift of three. Therefore, the reduced-set recommendation should be interpreted in relation to the operating regime, whereas full-proxy monitoring is the most trajectory-robust information-complete configuration.

**Figure 13:**
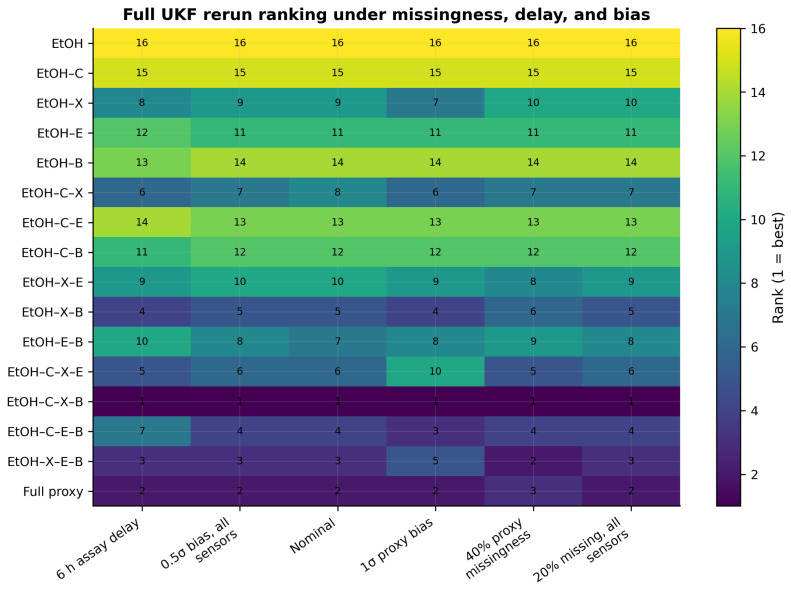
Sensor-set ranking for full UKF missingness, assay-delay, and measurement-bias stress tests. Rank 1 denotes the highest aggregate sensor-value score in each measurement-imperfection scenario.

Full UKF stress tests were then used to examine practical measurement imperfections, including missing observations, assay delay, and systematic measurement bias. In these tests, the complete UKF reconstruction and ranking workflow was rerun under each stress condition. The ethanol–sugar–biomass–substrate package remained top-ranked in all stress scenarios, as shown in Figure 13 and Table 11. The largest degradation in the top-package reconstruction error occurred in the 6h assay-delay case, where the mean latent-state RMSE of the top package increased to 0.5953. Systematic bias was injected as an additive measurement offset rather than treated only as zero-mean variance. These results show that the main aggregate recommendation remained stable when measurements were missing, delayed, or biased.

**Figure 14:**
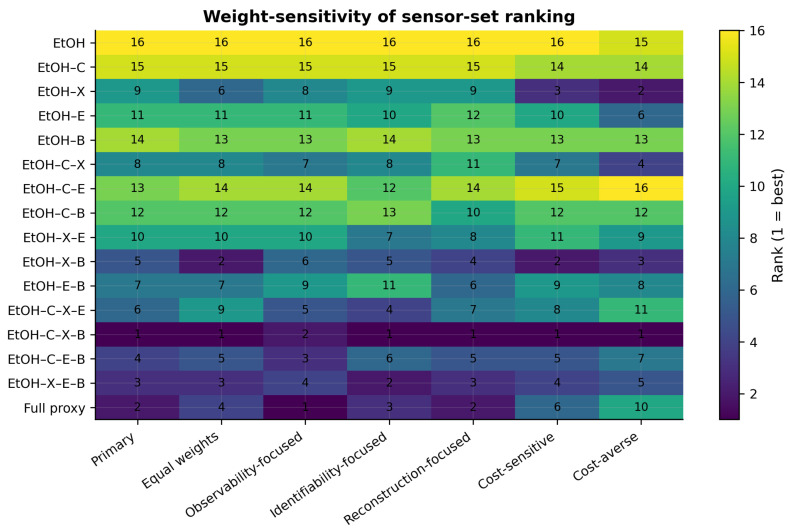
Sensor-set ranking under alternative scoring-weight assumptions. Rank 1 denotes the preferred sensor package for the corresponding design objective.

The weight-sensitivity analysis showed that the preferred package was also stable across most scoring priorities, as shown in Figure 14 and Table 12. The ethanol–sugar–biomass–substrate package remained top-ranked with the primary, equal-weight, identifiability-focused, reconstruction-focused, burden-sensitive, and burden-averse formulations. Full-proxy monitoring became top-ranked only when the observability term was given dominant weight. This confirms that the top-ranked reduced package is not merely a consequence of a single arbitrary weight choice, although full-proxy monitoring remains the preferred option when the main objective is maximum state-observability coverage.

Finally, alternative sensor-specific measurement-burden scenarios were tested to distinguish weight sensitivity from changes in the assumed practical workflow. The ethanol–sugar–biomass–substrate package remained top-ranked in all tested burden workflows, as summarized in Table 13. This suggests that the primary recommendation is not solely caused by the nominal burden values, although the relative ranks of nearby packages still changed when biomass, enzyme, spectroscopy, or solids-measurement assumptions were altered.

### 4.7. Implications for Practical CBP Digital-Twin Development

Within the tested five-state virtual-plant benchmark, the results support a hierarchical approach to measurement selection for CBP digital-twin development. Ethanol-only monitoring was consistently the weakest configuration because ethanol is a delayed product signal and cannot indicate whether limited batch performance originates from biomass limitation, enzyme insufficiency, substrate scarcity, slow hydrolysis, sugar accumulation, or product inhibition. This interpretation is consistent with bioprocess soft-sensing studies showing that delayed quality indicators and hidden physiological states can limit real-time monitoring and control [8,9,10,11,42].

Adding soluble sugar provided a small, low-burden improvement because sugar links substrate hydrolysis to ethanol formation. However, the ethanol–sugar package should be viewed as a minimal measurement configuration rather than a complete digital-twin sensor package. It does not directly capture biomass growth, enzyme activity, or residual substrate availability. This agrees with process analytical technology and digital-bioprocessing approaches, where intermediate measurements are informative but often need to be combined with soft sensors, model-based estimation, and digital-twin architectures [11,19,22,24,45,46].

The all-combination analysis changed the practical recommendation relative to the original restricted seven-package comparison. Across all 16 ethanol-mandatory combinations, ethanol–sugar–biomass–substrate achieved the highest primary aggregate score. This package combines a product signal, a fermentable-intermediate signal, a biological-state proxy, and a hydrolysis or solids-state proxy. It therefore provides strong state observability and UKF reconstruction while avoiding the highest burden of full-proxy monitoring. Full-proxy monitoring remains preferred when maximum reconstruction accuracy, state-observability coverage, and information completeness are required. The ethanol–sugar–biomass–enzyme package remains important for parameter learning because biomass and enzyme measurements provide strong information about growth, enzyme-production, and hydrolysis-related parameter directions, but it is no longer the strongest aggregate reduced package once residual-substrate-containing combinations are included.

In practice, low-burden screening experiments could begin with ethanol, sugar, and biomass measurements. Experiments focused on overall digital-twin readiness should add a residual-substrate or solids-related proxy, giving the ethanol–sugar–biomass–substrate package. Model-refinement experiments that prioritize kinetic identifiability should include enzyme activity, especially when growth and hydrolysis parameters must be separated. High-quality benchmark experiments should use full-proxy monitoring when the measurement burden is acceptable. This staged development route is consistent with digital bioprocessing and digital chemical engineering roadmaps, in which digital twins evolve through improved modeling, state estimation, process analytics, enabling digital technologies, and control-oriented decision support [19,20,21,23,24,25,45,46].

These practical implications should be interpreted as design guidance for the tested virtual-plant benchmark, not as a universal sensor prescription. Platform-specific sensor errors, costs, delays, organisms, feedstocks, product portfolios, missing-data patterns, and operating regimes should be incorporated before implementation. This is especially important for literature-derived CBP datasets, where product prediction can be affected by heterogeneous feedstock–pretreatment–microbial descriptors, sparse reporting, and missing-label structure [30].

### 4.8. Limitations

Several limitations apply to this study. First, a computational virtual plant was used to generate observations and evaluate sensor-set performance. The results therefore provide guidance for sensor prioritization and digital-twin readiness assessment, but they should not be interpreted as experimental validation. Real CBP systems may include organism-specific regulation, feedstock heterogeneity, mass-transfer limitations, inhibitor formation, contamination, evaporation, sensor drift, and assay-specific bias. Future work should test the proposed hierarchy using synchronized experimental measurements of ethanol, soluble sugars, biomass proxies, enzyme activity, and residual solids.

Second, the five-state hybrid model is a compact representation of lignocellulosic CBP. It captures the main information pathways needed to study state observability, parameter identifiability, and soft-sensor reconstruction, but it does not resolve all biochemical details. More detailed models could separate cellulose, hemicellulose, glucose, xylose, cellobiose, individual enzyme classes, inhibitor species, co-products, and organism-specific metabolic states. This extension is relevant because literature-derived CBP datasets show uneven product support and missing-label structure across ethanol and co-products [30]. Such refinements may change the relative importance of candidate sensors and may introduce additional identifiability challenges unless richer measurements are available [28,29].

Third, the observability and identifiability criteria are local and depend on the operating trajectory around which sensitivities are evaluated. This study examined nominal, mild, hydrolysis-extended, fermentation-early, and shifted-feasible temperature–pH trajectories. The overall ranking was highly correlated across these cases, but the top aggregate package changed for some trajectories. The ethanol–sugar–biomass–substrate package was preferred in the nominal and hydrolysis-extended cases, whereas full-proxy monitoring became preferred for the mild, fermentation-early, and shifted-feasible profiles. Therefore, the recommendation should be interpreted as conditional on the explored operating region. Other feedstocks, organisms, pretreatment severities, solids loadings, batch durations, or control policies may generate different sensitivity patterns. This is a general limitation of sensitivity-based experimental design and practical identifiability analysis [37,38].

Fourth, the measurement-noise levels, missingness assumptions, bias levels, assay-delay approximation, and measurement-burden indices were chosen as computational design scenarios rather than calibrated values for a specific experimental platform. The ranking was stable with uniform noise scaling, missing observations, assay delay, systematic bias, alternative scoring weights, and alternative burden workflows. However, the sensor-specific noise Monte Carlo analysis showed that full-proxy monitoring and the ethanol–sugar–biomass–substrate package can exchange the top rank when individual sensor uncertainties vary independently. Actual measurements can also have platform-specific error structures, detection limits, sampling losses, maintenance requirements, operator-time costs, and latency constraints. Future studies should replace the abstract burden and error assumptions with experimentally measured values for the intended laboratory or pilot-plant platform [21,23,42].

Fifth, the expanded analysis evaluated all ethanol-mandatory combinations of the five modeled measurement channels, but it did not evaluate sensor packages that omit ethanol. Ethanol was treated as mandatory because it is the direct product signal and the practical product-monitoring baseline. Therefore, the ranking should be interpreted within the ethanol-mandatory design space. Other objectives, such as early-stage hydrolysis diagnostics before ethanol formation, could motivate a different candidate space.

Sixth, the UKF case study evaluated latent-state reconstruction under model–plant mismatch conditions, but it did not test closed-loop control performance in an experimental process. Good estimator performance is only one requirement for digital-twin deployment. Practical implementation also requires suitable actuators, acceptable measurement latency, robust controller design, reliable data transfer, and safe interaction among the physical process, model updates, operators, and controllers. The present framework should therefore be viewed as a sensor-prioritization and soft-sensing readiness tool, not as complete closed-loop digital-twin validation [45,46].

Finally, the aggregate ranking depends on the normalized multi-criteria scoring scheme. Although the weight-sensitivity analysis showed that the ethanol–sugar–biomass–substrate package remained preferred under most weighting schemes, full-proxy monitoring became preferred when state observability was given dominant weight. The preferred package therefore depends on whether the digital twin is intended for maximum state-information coverage, parameter learning, nonlinear reconstruction, or lower-burden screening. The ranking should be used as a decision-support tool rather than a universal sensor prescription.

## 5. Summary and Conclusions

This paper presented a computational methodology for selecting informative measurement packages for digital-twin-assisted consolidated bioprocessing (CBP). The framework combines state-observability analysis, parameter-identifiability analysis, UKF-based soft-sensor reconstruction, measurement-burden assessment, and robustness testing with changes in measurement noise, operating trajectory, measurement imperfections, scoring weights, and sensor-specific burden assumptions. The objective was to support pre-experimental sensor-set design before laboratory or pilot-scale digital-twin validation.

Within the tested five-state virtual-plant benchmark, ethanol-only sensing was inadequate for state-aware CBP digital-twin reconstruction because ethanol is a delayed product signal. At a 6h sampling interval, the state-observability log-pseudodeterminant increased from 4.18 with ethanol-only sensing to 8.56 after adding soluble sugar and to 16.42 with full-proxy monitoring. The ethanol–sugar–biomass–substrate package also provided strong reduced state-observability performance, with log-pseudodeterminants of 15.12, 13.76, and 12.51 at 6, 12, and 24h, respectively. Parameter-identifiability analysis showed that biomass and enzyme proxies were especially valuable for model learning: the ethanol–sugar–biomass–enzyme package gave the strongest active-information performance, with log-pseudodeterminants of 10.82, 9.06, and 6.67 at 6, 12, and 24h, respectively. Full-proxy monitoring provided the most complete all-parameter information coverage.

The paired UKF Monte Carlo reconstruction test showed that additional measurements substantially improved latent-state estimation under model–plant mismatch conditions. Ethanol-only monitoring gave a mean latent-state RMSE of 1.1899, whereas full-proxy monitoring gave the lowest RMSE, 0.3756, followed by ethanol–biomass–enzyme–substrate at 0.3843 and ethanol–sugar–biomass–substrate at 0.4121. After evaluating all 16 ethanol-mandatory candidate packages, the aggregate ranking changed relative to the original seven-package comparison. Ethanol–sugar–biomass–substrate achieved the highest primary aggregate sensor-value score, 0.8432, with a burden index of 7.0. Full-proxy monitoring ranked second, with a score of 0.8173 and a burden index of 10.0, while ethanol–biomass–enzyme–substrate ranked third, with a score of 0.8086. The previously emphasized ethanol–sugar–biomass–enzyme package remained important for parameter learning but ranked sixth overall once residual-substrate-containing combinations were included.

The robustness analyses supported the main recommendation while clarifying its conditional nature. Ethanol–sugar–biomass–substrate remained top-ranked with uniform noise scaling, full UKF missingness, delay and bias stress tests, most scoring-weight scenarios, and all tested sensor-specific burden workflows. For independent sensor-specific noise variation, full-proxy monitoring and ethanol–sugar–biomass–substrate had similar top-rank frequencies, and for some alternative operating trajectories full-proxy monitoring became top-ranked. Overall, ethanol-only monitoring is suitable only as a minimal product baseline; ethanol–sugar–biomass sensing can support lower-burden screening; ethanol–sugar–biomass–substrate sensing is recommended for the primary aggregate digital-twin readiness score; ethanol–sugar–biomass–enzyme sensing remains attractive for parameter learning; and full-proxy monitoring is recommended for benchmark experiments when maximum reconstruction accuracy and information completeness are required. Because the results were obtained from a computational benchmark, the hierarchy should be validated with platform-specific experimental measurements, sensor errors, delays, costs, organisms, feedstocks, and operating regimes before practical deployment.

## Figures and Tables

**Table 1:** Nominal parameterization of the hybrid CBP virtual plant used for trajectory simulation, finite-difference sensitivity analysis, Fisher-information calculations, and UKF prediction. The values are computational benchmark assumptions, not organism-calibrated kinetic constants.

Quantity	Symbol or Expression	Nominal Value/Definition	Role
Batch duration	$t_{f}$	96h	Simulation horizon
Internal integration step	Δt	2h	RK4 integration step
Initial biomass	$X_{0}$	0.10	Initial active biomass state
Initial enzyme activity	$E_{0}$	0	Initial enzyme-activity state
Initial feedstock loading	$S_{0}$	100	Feedstock loading index
Initial insoluble substrate	$B_{0}=S_{0}/5$	20	Initial residual insoluble substrate
Initial soluble sugar	$C_{0}$	0	Initial soluble-sugar state
Initial ethanol	$P_{0}$	0	Initial ethanol state
Biomass carrying capacity	*K*	7.5	Logistic biomass-growth limit
Base growth-rate coefficient	$\mu_{0}$	$0.215\;h^{-1}$	Multiplies temperature, pH, and growth-scale factors
Base decay coefficient	$d_{0}$	$0.009\;h^{-1}$	Basal biomass-decay coefficient
Product-dependent decay factor	–	1+0.35max(0,P−6)/6	Increases biomass decay at elevated ethanol
Base enzyme-yield coefficient	$Y_{E,0}$	0.070	Enzyme-production coefficient
Enzyme degradation coefficient	$k_{\deg}$	$0.010+0.010\max{\left[0,\left(T-50\right)/10\right]}^{2}$	Temperature-dependent enzyme degradation
Base hydrolysis capacity	$V_{\max,0}$	0.235	Hydrolysis-capacity coefficient
Hydrolysis half-saturation term	$K_{m}$	$0.85/\max(0.35,f_{\mathrm{pret}}\theta_{\mathrm{feed}})$	Feedstock-accessibility-dependent hydrolysis term
Base ethanol-yield coefficient	$Y_{P,0}$	0.215	Fermentation coefficient
Base inhibition coefficient	$k_{\mathrm{inh},0}$	0.070	Ethanol-inhibition coefficient
Soluble-sugar loss term	–	0.10C	Non-fermentative soluble-sugar loss in the hydrolysis phase
Pretreatment factor	$f_{\mathrm{pret}}$	1.10 for nominal pretreatment index ≥0.5	Multiplies enzyme production and hydrolysis capacity
Nominal feedstock-accessibility scale	$\theta_{\mathrm{feed}}$	1.0	Multiplicative feedstock-accessibility factor
Multiplicative-factor bounds	–	$0.35\leq \theta_{i}\leq 2.75$	Numerical bounds for plant-scale factors
State bounds	–	0≤X,E≤20, 0≤B,C≤40, 0≤P≤30	Non-negativity and numerical clipping bounds
Operating bounds	–	30 °C≤T≤55 °C, 5.0≤pH≤8.0	Feasible input domain

**Table 2:** Sensor library for CBP sensor-set design. The burden index is a dimensionless relative measure of sampling effort, assay delay, calibration burden, and implementation difficulty.

Key	Measurement	σ	Burden Index
*P*	Ethanol	0.18	1.0
*C*	Sugar	0.20	1.5
*X*	Biomass proxy	0.05	2.0
*E*	Enzyme-activity proxy	0.06	3.0
*B*	Residual insoluble-substrate proxy	0.40	2.5

**Table 3:** Practical interpretation of the modeled CBP measurement channels.

Modeled Output	Possible Measurement Routes	Typical Implementation Mode	Main Practical Limitations
*P* Ethanol	HPLC, GC, enzymatic ethanol assay, NIR/Raman spectroscopy, ethanol biosensor	At-line or offline for chromatographic and enzymatic assays; potentially online for spectroscopy or biosensors	Assay or sampling delay, calibration transfer, spectral interference, sensor drift, and matrix effects in lignocellulosic broth.
*C* Soluble sugar	HPLC, enzymatic glucose/xylose assays, refractive-index methods, NIR/Raman spectroscopy	Mostly at-line or offline; spectroscopy may be online or in situ after calibration	Overlap among multiple sugars, matrix effects from hydrolysate components, calibration burden, and limited specificity for total fermentable sugar.
*X* Biomass proxy	Optical density, dry cell weight, capacitance/dielectric spectroscopy, turbidity, image analysis, soft-sensor estimate	Offline or at-line for dry weight and optical density; online possible for dielectric or turbidity probes	Interference from insoluble solids, bubbles, cell-morphology changes, fouling, and calibration dependence on organism and feedstock.
*E* Enzyme-activity proxy	Cellulase activity assay, fluorogenic or colorimetric enzyme assay, protein assay, soft-sensor estimate from hydrolysis response	Primarily at-line or offline	Assay latency, substrate specificity, enzyme-mixture complexity, inhibition effects, temperature and pH dependence, and limited online availability.
*B* Residual insoluble-substrate proxy	Gravimetric residual solids, total suspended solids, near-infrared spectroscopy, image analysis, solids-balance estimate	Mostly offline or at-line; online estimation possible through calibrated spectroscopy or soft sensing	Sampling heterogeneity, solids settling, poor representativeness, matrix effects, pretreatment-dependent calibration, and high measurement burden.

The table links the scalar measurement channels used in the computational model to plausible experimental or PAT implementations. The listed methods are not unique choices; they illustrate how each modeled output could be approximated in laboratory or pilot-scale CBP monitoring. The numerical noise and burden values used in the computational ranking are nominal workflow assumptions and should be replaced by platform-specific values when experimental sensor data become available.

**Table 4:** UKF Monte Carlo settings for reproducing the soft-sensing experiment.

Item	Setting
Candidate sets	16 ethanol-mandatory packages: {P}∪A, where A⊆{C,X,E,B}.
Base seed and pairing	Base seed =42. Plant mismatch and initial-estimate perturbations were indexed by replicate only, giving paired comparisons across sensor sets.
Simulation grid	$t_{f}=96\;h$, RK4 step =2h; errors evaluated at t=0,2,…,96h.
UKF sampling	Measurements every 12h, with updates at t=12,24,…,96h.
Initial covariance	$P_{0}=\mathrm{diag}{\left(0.06,0.08,3.00,0.60,0.40\right)}^{2}$, state order [X,E,B,C,P].
Process noise	$Q=\mathrm{diag}{\left(0.0099,0.0126,0.9000,0.03375,0.02925\right)}^{2}$.
Measurement noise	$R_{S}=\mathrm{diag}\left(\sigma_{i}^{2}\right)$, using the sensor noise values in Table 2.
Measurement-noise streams	Noise was generated by replicate, time, and channel; shared channels used the same noise draw across sensor sets.
Plant–model mismatch	Plant scales $s_{j}=\exp\left(\eta_{j}\right)$. For growth, enzyme yield, hydrolysis, ethanol yield, and feedstock: $\eta_{j}∼N\left(0,0.22^{2}\right)$. For decay and inhibition: $\eta_{j}∼N\left(0,{\left(0.65\times 0.22\right)}^{2}\right)$. Scales were clipped to [0.35,2.75].
Initial estimate	${\hat{x}}_{0}=x_{0}⊙\left(1+\epsilon \right)$, $\epsilon_{j}∼N\left(0,0.18^{2}\right)$, followed by state-bound clipping and ${\hat{B}}_{0}\geq 2$.
Measurement clipping	Noisy measurements were clipped to zero; bias offsets were added in bias-stress scenarios.
State bounds	0≤X,E≤20, 0≤B,C≤40, 0≤P≤30.
RMSE metric	Latent RMSE used X,E,B,C; ethanol was excluded because it was measured in all candidate sets.
Statistical comparison	2000 bootstrap resamples and 15 paired ethanol-only contrasts; Wilcoxon *p*-values were Holm- and Benjamini–Hochberg-adjusted.

**Table 5:** Main computational assumptions used in the sensor-set comparison.

Category	Values Used	Purpose
Sensor noise	$\sigma_{P}=0.18$, $\sigma_{C}=0.20$, $\sigma_{X}=0.05$, $\sigma_{E}=0.06$, $\sigma_{B}=0.40$	Weights FIM calculations and defines UKF measurement covariance.
Measurement burden	P=1.0, C=1.5, X=2.0, E=3.0, B=2.5	Represents relative sampling, delay, calibration, and implementation burden.
Candidate space	All 16 ethanol-mandatory combinations	Avoids ranking only a restricted seven-package list; ethanol is the product baseline.
Sampling intervals	6, 12, and 24h	Tests dense-to-sparse laboratory or pilot-scale sampling.
Finite differences	$\varepsilon_{j}=10^{-3}\max(|x_{0,j}\left|,1\right)+10^{-4}$; $\delta =10^{-3}$	Computes state and log-parameter sensitivities; clipped perturbations use the actual denominator.
FIM threshold	$\tau =\max(10^{-8}\lambda_{\max},10^{-12})$	Defines numerical rank and active eigenvalues.
Regularization	$\rho =\max(10^{-8}\lambda_{\max},10^{-10})$; $10^{-10}I$ for covariance pseudoinverse	Stabilizes determinant and uncertainty diagnostics.
Primary weights	$\left(w_{\mathrm{obs}},w_{\mathrm{id}},w_{\mathrm{ukf}},w_{\mathrm{burden}}\right)=\left(0.30,0.35,0.25,0.10\right)$	Balances observability, identifiability, reconstruction accuracy, and burden.
Alternative weights	Equal, observability-focused, identifiability-focused, reconstruction-focused, burden-sensitive, burden-averse	Tests dependence on design priorities.
UKF settings	α=0.35, β=2, κ=0; RK4 propagation	Common nonlinear filtering setup for all sensor sets.
Monte Carlo size	$N_{\mathrm{MC}}=100$ per candidate set	Estimates reconstruction-error distributions under mismatch and noise conditions.
Mismatch design	Multiplicative perturbations in growth, enzyme yield, hydrolysis, ethanol yield, decay, inhibition, and feedstock accessibility	Tests reconstruction when the estimator uses the nominal model but the virtual plant differs.
Measurement stresses	Missingness, 6h delay, moderate all-sensor bias, larger proxy bias	Reruns the complete UKF workflow under imperfect practical measurement conditions.
Noise robustness	200 sensor-specific noise scenarios with $m_{k}\in \left[0.5,2.0\right]$	Tests nonuniform changes in sensor accuracy.
Trajectory robustness	Nominal, mild, hydrolysis-extended, fermentation-early, shifted-feasible	Tests dependence on the temperature–pH excitation schedule.
Burden workflows	Nominal, spectroscopy/biosensor, offline assay, dielectric-biomass, high-solids burden	Tests sensitivity to sensor-specific implementation assumptions.

**Table 6:** Representative mean UKF RMSE values by sensor set.

Sensor Set	*X*	*E*	*B*	*C*	*P*
EtOH	1.01	0.45	3.02	0.28	0.16
EtOH–C	0.97	0.43	2.98	0.19	0.14
EtOH–B	0.99	0.44	1.13	0.27	0.16
EtOH–C–B	0.97	0.43	1.11	0.18	0.14
EtOH–C–X–E	0.12	0.08	3.43	0.19	0.16
EtOH–C–X–B	0.11	0.22	1.11	0.20	0.16
EtOH–X–E–B	0.12	0.08	1.11	0.23	0.17
Full	0.12	0.08	1.11	0.19	0.16

*X*: biomass, *E*: enzyme activity, *B*: substrate, *C*: sugar, *P*: ethanol. The table reports representative packages; the full 16-set RMSE distributions are shown in Figure 9.

**Table 7:** Paired comparison of latent-state UKF RMSE against ethanol-only monitoring.

Sensor Set	RMSE	Mean Red.	Bootstrap 95% CI	Holm *p*
EtOH–C–X–B	0.4121	0.7778	[0.7022,0.8558]	$5.84\times 10^{-17}$
Full proxy	0.3756	0.8143	[0.7398,0.8894]	$5.84\times 10^{-17}$
EtOH–X–E–B	0.3843	0.8056	[0.7305,0.8824]	$5.84\times 10^{-17}$
EtOH–C–E–B	0.4634	0.7265	[0.6516,0.8032]	$5.84\times 10^{-17}$
EtOH–X–B	0.4243	0.7656	[0.6883,0.8380]	$5.84\times 10^{-17}$
EtOH–C–X–E	0.9554	0.2345	[0.1619,0.3070]	$4.02\times 10^{-7}$
EtOH–E–B	0.4718	0.7181	[0.6434,0.7888]	$5.84\times 10^{-17}$
EtOH–C–X	1.0093	0.1806	[0.0997,0.2603]	$5.94\times 10^{-5}$
EtOH–X	0.9396	0.2504	[0.1884,0.3122]	$2.33\times 10^{-10}$
EtOH–X–E	0.8869	0.3030	[0.2446,0.3641]	$2.62\times 10^{-14}$
EtOH–E	0.9732	0.2167	[0.1574,0.2811]	$6.45\times 10^{-9}$
EtOH–C–B	0.6737	0.5162	[0.4509,0.5826]	$6.82\times 10^{-17}$
EtOH–C–E	1.0392	0.1507	[0.0727,0.2224]	$3.82\times 10^{-4}$
EtOH–B	0.7051	0.4848	[0.4174,0.5561]	$5.84\times 10^{-17}$
EtOH–C	1.1398	0.0501	[0.0144,0.0878]	$2.30\times 10^{-2}$

Baseline ethanol-only mean latent-state RMSE = 1.1899. Mean red. denotes the mean paired reduction in latent-state RMSE relative to ethanol-only monitoring. Bootstrap intervals used 2000 resamples. Holm-adjusted *p*-values were calculated over 15 ethanol-only contrasts. *C*: sugar, *X*: biomass, *E*: enzyme, *B*: substrate.

**Table 8:** Primary sensor-set ranking across all 16 ethanol-mandatory candidates.

Rank	Sensor Set	Burden	Score	Value/Burden
1	EtOH–C–X–B	7.0	0.8432	0.1205
2	Full proxy	10.0	0.8173	0.0817
3	EtOH–X–E–B	8.5	0.8086	0.0951
4	EtOH–C–E–B	8.0	0.7557	0.0945
5	EtOH–X–B	5.5	0.7395	0.1344
6	EtOH–C–X–E	7.5	0.7228	0.0964
7	EtOH–E–B	6.5	0.6573	0.1011
8	EtOH–C–X	4.5	0.6531	0.1451
9	EtOH–X	3.0	0.6465	0.2155
10	EtOH–X–E	6.0	0.6457	0.1076
11	EtOH–E	4.0	0.5846	0.1461
12	EtOH–C–B	5.0	0.4665	0.0933
13	EtOH–C–E	5.5	0.3892	0.0708
14	EtOH–B	3.5	0.3540	0.1012
15	EtOH–C	2.5	0.2894	0.1157
16	EtOH	1.0	0.1440	0.1440

EtOH: ethanol, *C*: sugar, *X*: biomass, *E*: enzyme, *B*: substrate. Burden is the dimensionless measurement-burden index. The rank is based on the primary aggregate sensor-value score. Value/burden is reported only as a secondary diagnostic and was not used to determine the rank. Latent-state RMSE values are reported separately in Table 6.

**Table 9:** Uniform noise-sensitivity summary. The top-ranked set was EtOH–C–X–B in all noise scenarios.

Noise Scenario	Multiplier	Top Score	Spearman ρ
Low noise	0.5	0.8450	0.9971
Nominal noise	1.0	0.8432	1.0000
High noise	2.0	0.8409	0.9882

EtOH–C–X–B denotes ethanol–sugar–biomass–substrate. Spearman ρ is relative to the nominal-noise ranking. Scores are normalized within each noise scenario.

**Table 10:** Operating-trajectory robustness summary.

Trajectory	Top Sensor Set	Top Score	Spearman ρ
Nominal	EtOH–C–X–B	0.8432	1.0000
Mild profile	Full proxy	0.8994	0.9941
Hydrolysis extended	EtOH–C–X–B	0.8465	0.9971
Fermentation early	Full proxy	0.8993	0.9912
Shifted feasible	Full proxy	0.8991	0.9647

Spearman ρ is relative to the nominal-trajectory ranking. Scores are normalized within each trajectory scenario.

**Table 11:** Full UKF missingness, delay, and bias stress-test summary. The top-ranked set was EtOH–C–X–B in all scenarios.

Stress Scenario	Top RMSE	Top Score	Spearman ρ
Nominal	0.4121	0.8432	1.0000
20% missing, all sensors	0.4683	0.8410	0.9941
Proxy missingness	0.5495	0.8321	0.9794
6h assay delay	0.5953	0.8483	0.9559
Moderate bias	0.4210	0.8430	0.9971
Proxy bias	0.4396	0.8506	0.9529

EtOH–C–X–B denotes ethanol–sugar–biomass–substrate. Spearman ρ is relative to the nominal stress-test ranking. Scores are normalized within each stress scenario.

**Table 12:** Top-ranked sensor sets under alternative scoring-weight assumptions.

Weighting Scheme	Top Sensor Set	Top Score	Burden
Primary	EtOH–C–X–B	0.8432	7.0
Equal weights	EtOH–C–X–B	0.7621	7.0
Observability focused	Full proxy	0.8520	10.0
Identifiability focused	EtOH–C–X–B	0.8352	7.0
Reconstruction focused	EtOH–C–X–B	0.8629	7.0
Burden sensitive	EtOH–C–X–B	0.7310	7.0
Burden averse	EtOH–C–X–B	0.6763	7.0

EtOH: ethanol, *C*: sugar, *X*: biomass, *E*: enzyme, *B*: substrate. Scores are normalized within each weighting scenario.

**Table 13:** Sensor-specific burden-workflow sensitivity summary. The top-ranked set was EtOH–C–X–B in all burden workflows.

Burden Workflow	Top Score	Spearman ρ
Nominal	0.8432	1.0000
Online spectroscopy/biosensor	0.8471	0.9912
Offline HPLC/assay workflow	0.8420	0.9971
Dielectric biomass available	0.8471	0.9824
High-solids burden	0.8367	0.9765

EtOH–C–X–B denotes ethanol–sugar–biomass–substrate. Spearman ρ is relative to the nominal burden-workflow ranking.

## Data Availability

The simulation code and numerical output files used to generate the figures and tables are available and have been archived in Zenodo at https://doi.org/10.5281/zenodo.20560602.

## Abbreviations

The following abbreviations are used in this study:

CBP	Consolidated bioprocessing
DT	Digital twin
FIM	Fisher information matrix
GC	Gas chromatography
HPLC	High-performance liquid chromatography
MAE	Mean absolute error
MC	Monte Carlo
NIR	Near-infrared spectroscopy
PAT	Process analytical technology
pdet	Pseudo-determinant
RK4	Fourth-order Runge–Kutta method
RMSE	Root mean squared error
UKF	Unscented Kalman filter
